# Existence of Bov-B LINE Retrotransposons in Snake Lineages Reveals Recent Multiple Horizontal Gene Transfers with Copy Number Variation

**DOI:** 10.3390/genes11111241

**Published:** 2020-10-22

**Authors:** Weerada Puinongpo, Worapong Singchat, Supaporn Petpradub, Ekaphan Kraichak, Mitsuo Nunome, Nararat Laopichienpong, Ratchaphol Thongchum, Thanphong Intarasorn, Siwapech Sillapaprayoon, Chantra Indananda, Narongrit Muangmai, Sunutcha Suntrarachun, Sudarath Baicharoen, Lawan Chanhome, Surin Peyachoknagul, Kornsorn Srikulnath

**Affiliations:** 1 Laboratory of Animal Cytogenetics and Comparative Genomics (ACCG), Department of Genetics, Faculty of Science, Kasetsart University, Bangkok 10900, Thailand; apple.wrd@gmail.com (W.P.); worapong.si@ku.th (W.S.); supaporn.pe@ku.th (S.P.); nararat.l@ku.th (N.L.); ratchaphol.t@ku.th (R.T.); siwapech.s@ku.th (S.S.); chantra007@yahoo.com (C.I.); fscisrp@ku.ac.th (S.P.); 2 Special Research Unit for Wildlife Genomics (SRUWG), Department of Forest Biology, Faculty of Forestry, Kasetsart University, Chatuchak, Bangkok 10900, Thailand; 3 Department of Botany, Faculty of Science, Kasetsart University, Bangkok 10900, Thailand; ekaphan.k@ku.th; 4 Laboratory of Animal Genetics, Department of Applied Molecular Biosciences, Graduate School of Bioagricultural Sciences, Nagoya University, Nagoya 464-8601, Japan; mtnunome@agr.nagoya-u.ac.jp; 5 Forensic DNA Division, Central Institute of Forensic Science, Ministry of Justice, Bangkok 10330, Thailand; atmapy@hotmail.com; 6 Department of Fishery Biology, Faculty of Fisheries, Kasetsart University, Bangkok 10900, Thailand; ffisnrm@ku.ac.th; 7 Queen Saovabha Memorial Institute, The Thai Red Cross Society, Bangkok 10330, Thailand; sunutcha@yahoo.com (S.S.); lchanhome@yahoo.com (L.C.); 8 Bureau of Conservation and Research, Zoological Park Organization under the Royal Patronage of His Majesty the King, Bangkok 10800, Thailand; sbaicharoen@yahoo.com; 9 Center for Advanced Studies in Tropical Natural Resources, Kasetsart University, Thailand (CASTNAR, NRU-KU), Bangkok 10900, Thailand; 10 Center of Excellence on Agricultural Biotechnology: (AG-BIO/PERDO-CHE), Bangkok 10900, Thailand; 11 Amphibian Research Center, Hiroshima University, Higashihiroshima 739-8526, Japan

**Keywords:** Bov-B LINE, copy number, horizontal transfer, retrotransposons, snake

## Abstract

Transposable elements (TEs) are dynamic elements present in all eukaryotic genomes. They can “jump” and amplify within the genome and promote segmental genome rearrangements on both autosomes and sex chromosomes by disruption of gene structures. The Bovine-B long interspersed nuclear element (Bov-B LINE) is among the most abundant TE-retrotransposon families in vertebrates due to horizontal transfer (HT) among vertebrate lineages. Recent studies have shown multiple HTs or the presence of diverse Bov-B LINE groups in the snake lineage. It is hypothesized that Bov-B LINEs are highly dynamic and that the diversity reflects multiple HTs in snake lineages. Partial sequences of Bov-B LINE from 23 snake species were characterized. Phylogenetic analysis resolved at least two Bov-B LINE groups that might correspond to henophidian and caenophidian snakes; however, the tree topology differed from that based on functional nuclear and mitochondrial gene sequences. Several Bov-B LINEs of snakes showed greater than 80% similarity to sequences obtained from insects, whereas the two Bov-B LINE groups as well as sequences from the same snake species classified in different Bov-B LINE groups showed sequence similarities of less than 80%. Calculation of estimated divergence time and pairwise divergence between all individual Bov-B LINE copies suggest invasion times ranging from 79.19 to 98.8 million years ago in snakes. Accumulation of elements in a lineage-specific fashion ranged from 9 × 10^−6^% to 5.63 × 10^−2^% per genome. The genomic proportion of Bov-B LINEs varied among snake species but was not directly associated with genome size or invasion time. No differentiation in Bov-B LINE copy number between males and females was observed in any of the snake species examined. Incongruence in tree topology between Bov-B LINEs and other snake phylogenies may reflect past HT events. Sequence divergence of Bov-B LINEs between copies suggests that recent multiple HTs occurred within the same evolutionary timeframe in the snake lineage. The proportion of Bov-B LINEs varies among species, reflecting species specificity in TE invasion. The rapid speciation of snakes, coinciding with Bov-B LINE invasion in snake genomes, leads us to better understand the effect of Bov-B LINEs on snake genome evolution.

## 1. Introduction

Transposable elements (TEs) are repeated DNA sequences that have the ability to move within a genome. In eukaryotes, TEs are often an abundant component of the genome [1,2,3]. Different classes of TEs are generally present in the same genome with diverse effects, such as promotion of genomic rearrangements or disruption of gene structures [4,5,6]. Comparison of nucleotide sequences of the same TE class or family reveals that TE copies show predominantly high nucleotide sequence similarities within species but low similarities among species [7,8,9]. By contrast, several TEs show high sequence similarities among distantly related species, which might result from interspecific transmission of TEs, a process termed horizontal transfer (HT) [9,10]. This might also indicate that evidence for HT is the lack of TE copies in the sister-species of the same evolutionary clade. This likely leads to the evolution of novel gene functions that assist in driving genetic variation [6,11,12]. More than half of the HTs documented in vertebrate genomes are derived from retrotransposons, and this might affect genome evolution [9,13]. Retrotransposons have the innate ability, using a copy-and-paste mechanism via an RNA intermediate, to jump within or between species, resulting in massive amplification of copy number variation and subsequent rapid increase in genome size over a short period in some lineages [1,14,15]. Retrotransposons amplified drastically in ruminants, while in horses, they retained low copy number of retrotransposons. This might result from different host silencing mechanisms [16]. Interestingly, the Bovine-B long interspersed nuclear element (Bov-B LINE) is among the most abundant retrotransposon families in vertebrates that show evidence of HT [17,18,19]. Bov-B LINEs were originally identified in cow, and their nucleotide sequences were observed to be closely related to those of bovids, including goats, sheep, and buffalo, leading to detection of a lineage-specific Bov-B LINE in Ruminantia [20,21,22,23]. Similarly, highly conserved Bov-B LINEs have been detected in squamate reptiles [24]. Comparison of Bov-B LINE sequences of many insects and vertebrates has revealed that ixodid ticks (reptile ticks) might be vectors for the transfer of BoV-B LINEs among vertebrate lineages. The transferred sequences are subsequently amplified, mutated, and diversified into lineage-specific Bov-B LINEs [17]. However, a lineage-specific Bov-B LINE has not been detected in snakes. The Bov-B LINEs of python and rat snakes are placed in different clades from those of viper and king cobra mixed with mammalian Bov-B LINE sequences [19]. This finding leads to the hypothesis that Bov-B LINEs are highly dynamic, and multiple HT events involving Bov-B LINEs have occurred in the snake lineage, in contrast to other vertebrates. However, the evolutionary history of Bov-B LINEs within multiple snake species has not been well studied [19,24].

Two groups of snakes are recognized at infraorder rank: (i) Scolecophidia, commonly termed “blind” snakes, and (ii) Alethinophidia, comprising Henophidia (pythons, boas, and other “primitive” snakes) and Caenophidia (advanced snakes). Snake genomes are relatively small, with an average DNA content of about 1.45 Gb [25,26,27,28,29,30,31,32]; however, recent snake genomic analyses have revealed that the remarkable variation in genome size reflects substantial differences in the amount of repeated DNA, such as satellite DNA (satDNA) and TEs [32,33]. The Z or the W sex chromosomes of several advanced snakes contain large numbers of satDNA and TE copies [32,34,35,36,37]. This finding suggests a correlation between repeated DNA and the differentiation process for snake sex chromosomes [32,37,38,39,40,41]. Snakes thus represent an ideal model for investigating TE dynamics in the genome, including HT and copy number variation concomitantly. However, relatively little is known about Bov-B LINE distribution in snakes because relatively few snake reference genomes are publicly available [25,26,27,28,30,31,32,42]. For an improved understanding of snake Bov-B LINE sequence evolution, it is essential to adopt a targeted and systematic experimental approach to investigate TE sequences in multiple snake species. In the present study, by taking advantage of this sequence similarity and copy number variation, we sought to test the following five hypotheses on the evolutionary origin of Bov-B LINEs: (i) Bov-B LINE originated in the genome of a common snake ancestor and was subsequently either inherited in certain lineages or lost in others, (ii) multiple HTs of Bov-B LINE independently occurred in different evolutionary lineages of snakes, (iii) copy number variation of Bov-B LINE occurred independently in different evolutionary lineages of snakes, (iv) variation in Bov-B LINE copy number among snakes depends on invasion time, and (v) copy number variation of Bov-B LINE among snakes is associated with the degree of differentiation in snake sex chromosomes. We first screened 23 snake species for the presence of Bov-B LINE using molecular cloning and sequencing of reverse transcriptase (RT) gene sequences. This method has previously been used to detect the status of HTs in phylogenetic analyses of metazoans [7,43,44]. In addition, Bov-B LINE copies in the snake genome were quantified using quantitative real-time polymerase chain reaction (qPCR) and determined in silico for the copy number of Bov-B LINE in entire genome sequences. The results provide an improved understanding of the evolutionary dynamics of Bov-B LINE in snake genomes.

## 2. Material and Methods

### 2.1. Specimen Collection and DNA Extraction

Detailed information is presented in Table 1. All snake samples, comprising 23 species from nine families, were collected from the Queen Saovabha Memorial Institute (Bangkok, Thailand) and Real Zoo (Ayutthaya, Thailand). The sex of each individual was determined from morphology and confirmed using a molecular sexing approach [45,46,47]. Blood samples were collected from the ventral tail vein using a 23 gauge needle attached to 2 mL disposable syringes. The syringes contained 10 mM ethylenediaminetetraacetic acid for DNA extraction. Total genomic DNA was extracted, following the standard salting-out protocol as described previously by Supikamolseni et al. [48], and used as templates for PCR amplification. Animal care and all experimental procedures were approved by the Animal Experiment Committee, Kasetsart University, Thailand (approval no. ACKU59-SCI-034 and ACKU61-SCI-024) and conducted in accordance with the Regulations on Animal Experiments at Kasetsart University.

### 2.2. Polymerase Chain Reaction Amplification and Molecular Cloning of Bov-B LINE

Generally, all the copies of Bov-B LINE found in different vertebrates do not exceed a length of about 3.1 kb. Based on the most conservative sequence of reverse transcriptase (RT) domain, all the LINEs are classified into several clades [49]. DNA fragments of Bov-B LINE sequences were amplified using primers specific for Bov-B LINE (BovB_VA2 F 5′-GCTACACTCAATTTGCCAGCA-3′ and BovB_VA2 R 5′- CCAGTTCTCCCTGTTGCTTC-3′). The primers were designed based on RT-specific Bov-B LINE domain sequences of *Vipera ammodytes* (accession number: AF332697.1), *Echis coloratus* (accession number: AF332673.1), and *Crotalus horridus* (accession numbers: AF332671.1, AF332672.1), which are available in the National Center for Biotechnology Information (NCBI) and the Repbase databases [50,51]. Because of the high cost of sequencing and the need to examine more snake species specimens, we performed sequence analysis at RT-specific Bov-B LINE domain (<400 bp). PCR amplification was performed using 15 μL of 1× ThermoPol buffer containing 1.5 mM MgCl_2_, 0.2 mM dNTPs, 5.0 μM of each primer, 0.5 U *Taq* polymerase (Apsalagen Co., Ltd., Bangkok, Thailand), and 25 ng genomic DNA. The PCR conditions were as follows: initial denaturation at 94 °C for 3 min, followed by 35 cycles of 94 °C for 30 s, 50 °C for 40 s, and 72 °C for 1 min 30 s, and final extension at 72 °C for 10 min. The PCR products were visualized by electrophoresis in 1% agarose gel and cloned molecularly using the pGEM-T Easy Vector (Promega Corporation, Madison, WI, USA). The nucleotide sequences of the DNA fragments were determined using the DNA sequencing services of First BASE Laboratories Sdn Bhd (Seri Kembangan, Selangor, Malaysia). The BLASTn (Nucleotide Basic Local Alignment Search Tool) program (http://blast.ncbi.nlm.nih.gov/Blast.cgi) was used to search for nucleotide sequences in the NCBI database to confirm the identity of the amplified DNA fragments. All sequences were deposited in the DNA Data Bank of Japan (DDBJ) (Tables S1 and S2). 

### 2.3. Survey of Bov-B LINE Copies in Publicly Released Snake Genome Assemblies

The FASTA genome sequences from nine snake species were retrieved from the NCBI website (http://www.ncbi.nlm.nih.gov) for Burmese python (*Python bivittatus*; AEQU00000000), garter snake (*Thamnophis sirtalis*; LFLD00000000), corn snake (*Pantherophis guttatus*; JTLQ01000000), king cobra (*Ophiophagus hannah*; AZIM00000000), European adder (*Vipera berus berus*, JTGP00000000), timber rattlesnake (*Crotalus horridus*; LVCR00000000.1), speckled rattlesnake (*Crotalus mitchellii pyrrhus*; JPMF01000000), habu snake (*Protobothrops flavoviridis*; BFFQ00000000.1), and Siamese cobra (*Naja kaouthia*, PRJNA506318) [25,26,27,28,29,30,31,32]. These genomes were assembled using different sequencing technologies and with various degrees of sequencing coverage and assembly effort. Scaffolds were determined for all genome sequences (*P. bivittatus*, *n* = 39,112; *T. sirtalis*, *n* = 7,930; *V. berus berus*, *n* = 25,713; *P. guttatus*, *n* = 883,920; *O. hannah*, *n* = 296,399; *C. horridus*, *n* = 186,068; *C. mitchellii pyrrhus*, *n* = 473,380; *P. flavoviridis*, *n* = 84,502; and *N. kaouthia*, *n* = 373,317). Alignment results with the input sequence as 330–385 bp were sorted manually using the BLASTN with default parameters. Bov-B LINE hits were initially identified in each genome using an iterative query-driven method based on sequence similarity. The consensus sequence of the RT domain of Bov-B LINE was used as the input query following a best-hits filtering algorithm using the default settings. This process was repeated three times to accommodate the inclusion of a new genome assembly version at various time stages in the pipeline. Fragments with BLAST hits that showed more than 80% (e-value 1 × 10^50^) identity and were longer than 325 bp were included in calculation of the copy number of Bov-B LINE.

Candidate sequences were obtained for each snake genome. A multiple sequence alignment of Bov-B LINE sequences was generated, and sequences were clustered into multiple groups using maximum likelihood (ML) analysis with 1000 replication bootstraps following the default parameters of Molecular Evolutionary Genetics Analysis 10 (MEGA X) software (Center for Evolutionary Functional Genomics, The Biodesign Institute, Tempe, AZ, USA) [52,53]. ML analysis was performed using the IQ-TREE algorithm [54] on the IQ-TREE webserver [55], and the TIM model was selected using the “auto” model selection option. We then selected representative candidate sequences from each major group for further analysis. To determine whether the sequence belonged to a previously described family, we compared it to the consensus sequences in the Repbase and the GenBank databases [56,57]. We considered a consensus sequence to be previously described if we detected a match of more than 98% nucleotidic identity with a described element in the same species.

### 2.4. Sequence Analysis

A multiple sequence alignment of Bov-B LINE sequences was generated with sequences from other vertebrates and insects sourced from the NCBI GenBank database and from the snake genome sequences, as mentioned above (Table 2), using the default parameters of MEGA X software [53]. All unalignable sites and gap-containing sites or missing data were carefully removed from the data set manually. Numbers of insertions and deletions (indels) were manually checked for each sequence of snake species using the multiallelic mode of DNAsp 6.0 [58]. Transitions and transversions were identified using the “strataG” function in R version 3.5.1 software [59]. Phylogenetic analysis was performed using maximum likelihood (ML) with the best-fit model and settings as mentioned above. Intraspecific nucleotide diversity (π value) among Bov-B LINE sequences at the clade level was analyzed using DNAsp 6.0. The degree of sequence divergence between species or between clades was estimated using uncorrected pair-wise distances (*p*-distances) as implemented in MEGA X [46]. Within- and between-families *p*-distance of the sequences were calculated using the function “rdist” in the package “rdist” in R [60]. Wilcoxon’s ranked-sign test was performed to compare the *p*-distances between and within families using the function “wilcox.test” in R [59,61]. Subsequently, Tukey’s honest significant difference test was used to detect genetic differentiation among Bov-B LINE families within and between clades, using the function “TukeyHSD” in R [62]. AMOVA [63] was performed to clarify the degree of genetic differentiation among clades by determining molecular variance and calculating *F*-statistics using ARLEQUIN version 2.000 with 1000 permutations [64]. For each species, nucleotide sequences were determined for at least four Bov-B LINE fragments amplified with the corresponding primer sets and/or derived from genome sequences, and the consensus sequences were generated based on the total alignment of units in each species using the BioEdit sequence alignment editor version 7.2.5 [65] by choosing the most frequent nucleotide at each position. Analysis of molecular variance (AMOVA) was also used to analyze the degree of genetic differentiation among species using ARLEQUIN 2.000 with 1000 permutations [64]. Synonymous (*K*_s_) and nonsynonymous (*K*_a_) substitution rates between species or between clusters and their standard errors were calculated in accordance with the Nei–Gojobori method [66] with Jukes–Cantor correction [67]. Simultaneously, we also tested whether the pattern of mutations observed between each copy, and the consensus sequence was significantly different from the expected value if the sequence had evolved neutrally, using the codon-based *Z*-test in MEGA X with the Nei–Gojobori method and Jukes–Cantor correction (1000 bootstrap replicates). 

### 2.5. Divergence Time Estimation and Mutation Rate between Bov-B LINE, COI, and BDNF

In addition to Bov-B LINE, pairwise distances between the snake species included in this study were calculated for *COI* and *BDNF* sequences. For *COI* and *BDNF*, distances were calculated between sequences generated during this study or available in GenBank for the species. DNA fragments of *COI* and *BDNF* sequences were amplified using gene-specific primers (COI-F: 5′-TCAGCCATACTACCTGTGTTCA- 3′ and COI-R: 5′-TAGACTTCTGGGTGGCCAAAGAATCA-3′ and BDNF-F: 5′-CAGCTTGGCTTATCCTGGTC-3′ and BDNF-R: 5′-CTTTGTGCTGCACTTGGTCTC-3′) [47,68]. PCR amplification was performed as mentioned above and in accordance with PCR protocols for each gene described by Tawichasri et al. [43] and Makowsky et al. [68]. The PCR products were visualized by electrophoresis in 1% agarose gel and molecularly cloned using the pGEM-T Easy Vector (Promega Corporation, Madison, WI, USA). Nucleotide sequences of the DNA fragments were determined using the DNA sequencing services of First BASE Laboratories Sdn Bhd (Seri Kembangan, Selangor, Malaysia). The BLASTn program (http://blast.ncbi.nlm.nih.gov/Blast.cgi) was used to search for nucleotide sequences in the NCBI database to confirm the identity of the amplified DNA fragments. All sequences were deposited in the DNA Data Bank of Japan (DDBJ) (Tables S1 and S2). Multiple sequence alignments of *COI* and *BDNF* sequences were generated using the default parameters of MEGA X software [53]. All unalignable sites and gap-containing sites or missing data were carefully removed from the data sets. 

The evolutionary rate and 95% highest posterior density (HPD) of the nucleotide sequences of the three genomic regions were estimated using BEAST 2.0 [69]. The analysis was conducted using the HKY85 substitution model and the strict molecular-clock model. Four calibration points were applied for *COI* and *BDNF* evolutionary rate estimation: 91.0 million years ago (MYA) for the time to the most recent common ancestor (TMRCA) of all sequences, 45 MYA for the TMRCA of Colubridae, 35 MYA for the TMRCA of Elapidae, and 24 MYA for the divergence time between *Enhydris* and *Homalopsis*. These divergence times were obtained from Vidal et al. [70]. We used two calibration points to estimate the *BovB* substitution rate: 64 MYA for the TMRCA of the sequences belonging to Colubroidea (Clade II in our phylogenetic tree), and 90 MYA for the TMRCA of the sequences belonging to Henophidia (Clade IA in our phylogenetic tree). Bayesian searches were conducted using the Markov chain Monte Carlo (MCMC) method for 30 million generations. The initial three million generations were discarded as the burn-in. Tracer version 1.6 [71] was used to check the convergence of the MCMC chains. The initial 25% saved trees were removed as the burn-in, and a maximum credibility tree based on the remaining trees was generated using TreeAnnotator version 2.0.2 (part of the BEAST version 2.0.2 package). A time-calibrated tree with 95% HPD was visualized and edited in FigTree version 1.3.1 [72]. Using a complete *COI* sequence (about 1000 bp) might be insufficient for estimations of deep divergences (>100 million years) [73]; however, we estimate the divergence time range based on *BDNF* and Bov-B LINE. A similar case of short size *COI* sequences was used to estimate divergence time in *Sitalcina sura* species group [74].

### 2.6. Quantification of Variation in Bov-B LINE Copy Number Based on Quantitative Real-Time Polymerase Chain Reaction (qPCR)

Quantification of Bov-B LINE in each snake species was performed by qPCR using an absolute quantification approach [75]. Bov-B LINE sequences were amplified using specific primers: F-BovB_VA2 and R-BovB_VA2. The qPCR amplification was performed using 10 μL of 2× KAPA SYBR^®^ FAST qPCR Master Mix (Kapa Biosystems, Cape Town, South Africa), 0.25 μM primers, and 25 ng genomic DNA. The PCR conditions were as follows: initial denaturation at 95 °C for 10 min, followed by 40 cycles of 95 °C for 15 s, 40–45 °C for 15 s, and 72 °C for 15 s, with a final extension at 72 °C for 5 min. A melting curve was plotted over a temperature range of 60 to 95 °C after each run to verify the absence of nonspecific product amplification. Amplification specificity was confirmed by dissociation curve analysis. In addition, specificity of the amplified product was examined by electrophoresis in 1% agarose gel. No template control was included in any run. Reactions were conducted in a 96-well optical plate, and a melt curve was generated to evaluate primer specificity. qPCR reactions of all specimens were performed with three technical replicates. For absolute quantification, a 10-fold serial dilution series of the clones (plasmid DNA harboring the Bov-B LINE clone) ranging from 1 × 10^9^ to 1 × 10^4^ was used to generate a standard curve (five-point serial dilutions) (Figure S1). The concentration of the recombinant plasmid was determined using NanoDrop™ 2000/2000c spectrophotometers (Thermo Fisher Scientific, Waltham, MA, USA). The plasmid copy number was calculated using the following equation: DNA (copy number) = [(6.023 × 10^23^) × (copy number/mol) × DNA amount (g)]/[DNA length (bp) × 660 × (g/mol/bp)], where Avogadro’s number = 6.023 × 10^23^ molecules (copy number/mol) with an average molecular weight of a double-stranded DNA molecule of 660 g/mol/bp. Total DNA length was 3465 bp (pGEM-T Easy Vector and inserted DNA (Bov-B LINE sequences) were 3015 and 450 bp, respectively). Cycle threshold (Ct) values in each dilution were measured using qPCR to create standard curves. A standard curve was generated by plotting Ct values against the log concentration of Bov-B LINE. To test intra-plate repeatability, the intra-assay coefficient of variation (CV) was measured in triplicate for plasmid DNA with the Bov-B LINE clone. The percentage CV for each sample was calculated by determining the standard deviation (SD) of each triplicate Ct value of Bov-B LINE, then dividing by the triplicate mean and multiplying by 100. Intra-assay percentage CVs should be less than 10 [76,77,78]. In the present study, the intra-assay CV was 1.2709–8.0354 for plasmid DNA harboring the Bov-B LINE clone. All samples fell within the concentration range generated by the standard curve. The copy number in the total DNA sample was determined by interpolating its Ct values against the standard curve. Absolute quantification was transformed into fold change values using the standard curve equation and compared with a reference sample. To test intra- and inter-plate repeatability, 113 snake samples were run in triplicate on four different plates to monitor plate-to-plate variation. The plate mean for each triplicate value was calculated and subsequently computed to determine overall mean, SD, and CV (%). Inter-assay CVs of less than 15% are generally acceptable [76,77,78]. In this study, the inter-assay CV (*n* = 113) was 2.37%, whereas that for the intra-assay CV was 2.22% for Bov-B LINE. Statistical differences in copy number among snake species were examined using the Kruskal–Wallis rank sum test implemented in the “stats” package in R [59]. Estimated values were expressed as the mean ± standard deviation. 

To examine differences in copy number of Bov-B LINE between male and female individuals, qPCR was performed using males and females (two or three individuals per species per sex) of 12 snake species (*Cylindrophis ruffus*, *Xenopeltis unicolor*, *Python bivittatus*, *Python regius*, *Acrochordus javanicus*, *Homalopsis buccata*, *Naja kaouthia*, *Naja siamensis*, *Bungarus candidus*, *Ahaetulla prasina*, *Coelognathus radiatus*, and *Ptyas mucosa*) from genomic DNA selected from the samples available. The PCR conditions and quantification were performed as described above. Statistical differences in copy number between male and female snakes were examined using a Wilcoxon signed-rank test implemented in the “stats” package in R [59]. 

## 3. Results

### 3.1. Characterization of Bov-B LINE

Specific Bov-B LINE primers were used to amplify RT-specific Bov-B LINE sequences, which yielded PCR products ranging in length from 420 to 450 bp. A total of 145 new nucleotide sequences of monomer units (or one copy unit of Bov-B LINE) were obtained with lengths ranging from 332 to 385 bp (Table 1). Several indels were detected and all Bov-B LINE sequences showed AT-bias with an average AT content of 58.6%. The ratio of transitions to transversions was 0.511. A BLASTn search of Bov-B LINE sequences in the NCBI database revealed similarities, ranging from 78.62% (*Xenopeltis unicolor* and *Vipera ammodytes* (AF332689.1) to 99.47% (*Crotalus horridus* (LVCR00000000.1) [30] and *Crotalus horridus* (AF332671.1)), with Bov-B LINEs isolated in previous studies [79]. No similarity was observed with other sequences deposited in the database. Partial sequences of Bov-B LINE were also identified in the genome sequences of seven snakes. Bov-B LINE sequences were mapped to scaffolds with the percentage of identical matches ≥ 80 and e-value ≤ 1e-50 in *Python bivittatus*, *Protobothrops flavoviridis*, *Vipera berus berus*, *Crotalus horridus*, *Crotalus pyrrhus*, *Naja kaouthia*, *Ophiophagus hannah*, *Thamnophis sirtalis*, and *Pantherophis guttatus*. Bov-B LINE sequences represented 3 × 10^−5^% to 1.98 × 10^−2^% of the snake genome (Table 2). We obtained 37 candidate sequences for *P. bivittatus*, 453 for *P. flavoviridis*, 639 for *V. berus berus*, 44 for *C. horridus*, 1 for *C. pyrrhus*, 89 for *N. kaouthia*, 99 for *O. hannah*, 30 for *T. sirtalis*, and 39 for *P. guttatus* from BLASTn searches of the respective genome assemblies. For each species, we clustered the sequence groups according to ML phylogenetic clade topology and constructed a consensus sequence. We thus obtained six consensus sequences for *P. bivittatus*, 10 for *P. flavoviridis*, 8 for *V. berus berus*, 6 for *C. horridus*, 1 for *C. pyrrhus*, 6 for *N. kaouthia*, 6 for *O. hannah*, 6 for *T. sirtalis*, and 6 for *P. guttatus*. All consensuses represented new families of Bov-B LINE. These results indicate that many of the identified sequences correspond to new elements from known families in snake genomes but have never previously been identified from the database. This implies that a large number of unidentified sequences remain to be discovered in the databases. 

**Figure 1:**
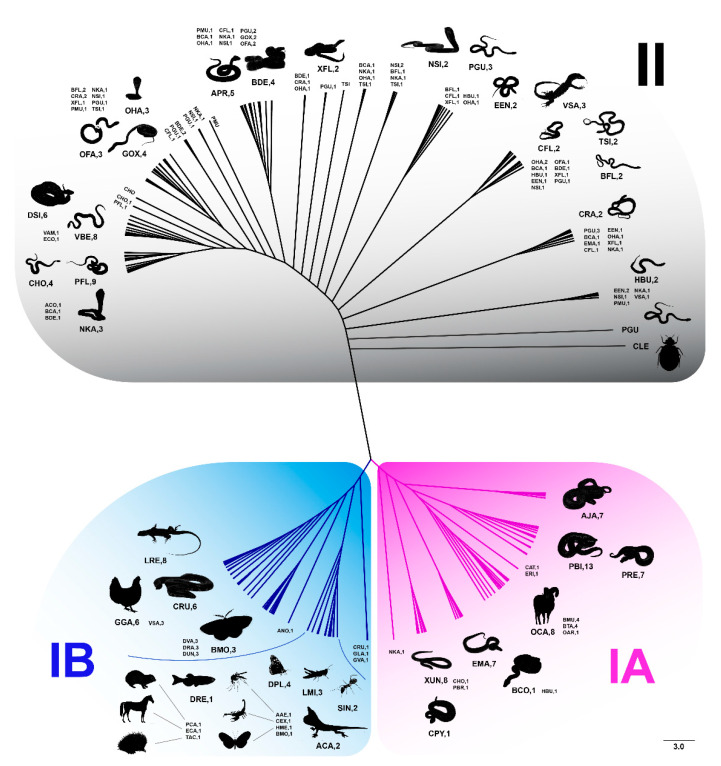
Phylogenetic relationships of Bov-B LINE retrotransposon sequences among eight snake families and outgroups inferred using maximum likelihood analysis. A colored line indicates different groups (Bov-B group I (with subgroups IA and IB) and group II). Bov-B LINE retrotransposon sequences of the snake lineages included Henophidian snakes (Cylindrophidae, Boidae, and Pythonidae families), Caenophidian snakes (Acrochordidae, Viperidae, Homalopsidae, Elapidae, and Colubridae families), and outgroups.

An ML phylogenetic tree was constructed to infer the evolutionary relationships among Bov-B LINE sequences from all snake lineages and to identify putative evolutionary Bov-B LINE groups using sequences from all snake species studied, other vertebrates, and insects. These groups were defined according to a set of specific nucleotide substitutions or indels. The Bov-B LINE sequences were grouped into two major clades. Many species were closely clustered in the ML tree despite being taxonomically unrelated, such as *C. pyrrhus*, *C. horridus*, *Homalopsis buccata*, and *N. kaouthia*, whereas several taxonomically closely related species showed no evidence of harboring similar Bov-B LINE copies, such as *Epicrates maurus* and the majority of caenopidian snakes (Figure 1 and Table S3). Group I contained two subgroups. Subgroup IA comprised 67 sequences from 10 snake species, of which the majority were henophidian snakes, with two scincid lizards and five mammalian species (Figure 1 and Table S3). Subgroup IB comprised 57 sequences from one snake species, *Cylindrophis ruffus*, seven insect species, nine lizard species, three mammalian species, chicken, and one fish species (Figure 1 and Table S3). Group II contained 158 sequences from 25 snake species; the majority were caenophidian snakes, with one bed bug and *Varanus salvator* (Figure 1). Remarkably, Bov-B LINE sequences of *V. salvator* were placed in subgroup IB and group II, and Bov-B LINE sequences of *E. maurus*, *C. horridus*, *H. buccata*, and *N. kaouthia* were classified in subgroup IA and group II.

### 3.2. Sequence Variability of Bov-B LINE within and between Snake Species

Average intraspecific sequence divergence (π) was 8.64 ± 0.05% (ranging from 4.00% ± 0.01 in *P. bivittatus* and *P. regius* to 14.00% ± 0.01 in *B. flaviceps*) (Table 3), whereas the average interspecific sequence divergence (*p*-distance) was 8.64% ± 0.01 (ranging from 0% ± 0.00 between *Echis coloratus* and *V. ammodytes* to 24% ± 0.06 between *Agkistrodon contortrix* and *Acrochordus javanicus*) (Table S4). Wilcoxon’s signed-rank test indicated that intra- and interspecific distances differed significantly (*p* < 0.05). Analysis of molecular variance (AMOVA) among Bov-B LINE sequences revealed significant variation among the species (*p* < 0.001, *F*_st_ = 0.19) and significant variation within a species (*p* < 0.001, *F*_st_ = 0.23). The average *K*_a_/*K*_s_ value of the Bov-B LINE sequences was 0.73 ± 0.26 (Table S5). Results of the *Z*-test showed that the number of nonsynonymous substitutions accumulated in Bov-B LINE copies since their insertion in snakes differed significantly from the number of synonymous substitutions (*Z*-test *p* < 0.05, stat = 1.74).

### 3.3. Sequence Variability within and between Bov-B LINE Groups

For data sets excluding the outgroup, the average π value of each Bov-B LINE group was 11% ± 0.01 for group I, 9% ± 0.00 for group II, 10% ± 0.01 for subgroup IA, and 7% ± 0.02 for subgroup IB (Table 4). Average sequence divergence between Bov-B LINE groups (*p*-distance) was 8.64% ± 0.01 (9% ± 0.01 between groups I and II, 9% ± 0.02 between subgroups IA and IB, 9% ± 0.01 between subgroup IA and group II, and 9% ± 0.02 between subgroup IB and group II) (Table 5). The average π value of each snake Bov-B LINE group with the outgroup included was 22% ± 0.02 for group I and 9% ± 0.00 for group II (Table 6). Average sequence divergence between Bov-B LINE groups (*p*-distance) was 15.32% ± 0.02 (18% ± 0.04 between groups I and II, 24% ± 0.04 between subgroups IA and IB, 12% ± 0.04 between subgroup IA and group II, and 24% ± 0.04 between subgroup IB and group II) (Table 7). Tukey’s honest significant difference test indicated that distances within subgroups IA and IB and between subgroups IA, IB, and group II were significantly different from each other (*p* < 0.001), whereas distances within subgroup IA and group II were not significantly different from each other (*p* = 0.79). AMOVA of Bov-B LINE sequences revealed significant variation among families (*p* < 0.001, *F*_st_ = 0.15) and significant variation within one family (*p* < 0.001, *F*_st_ = 0.34). Average *K*_a_/*K*_s_ values of Bov-B LINE sequences were 0.61 ± 0.17 and 0.77 ± 0.56 for groups I and II, respectively, and 0.55 ± 0.13 and 0.70 ± 0.19 for subgroups IA and IB, respectively. Results of the *Z*-test showed that the number of nonsynonymous substitutions accumulated in Bov-B LINE copies since their insertion in snakes was significantly different from the number of synonymous substitutions in each group (*Z*-test, *p* < 0.05, stat = 2.84). 

### 3.4. Comparison of Bov-B LINE Copy Numbers among Snake Species

**Figure 2:**
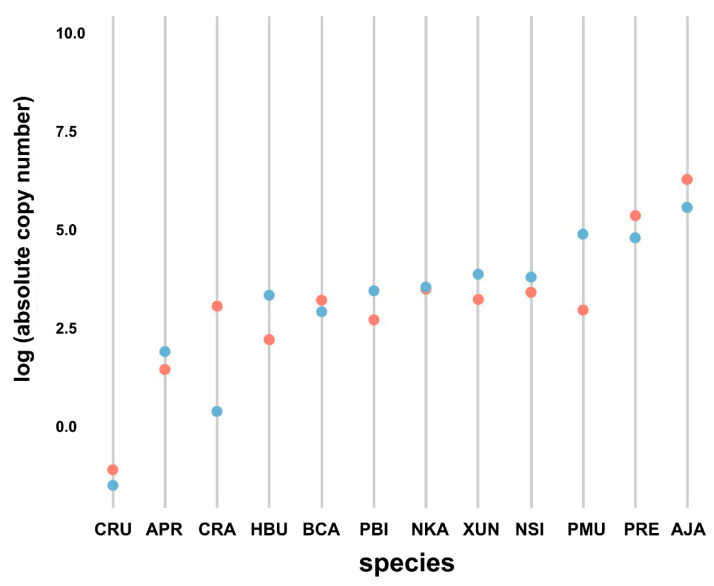
Accumulated copy number of *Bov-B* LINE retrotransposons in snake species determined by quantitative real-time PCR. *Cylindrophis ruffus* (CRU), *Ahaetulla prasina* (APR), *Coelognathus radiatus* (CRA), *Homalopsis buccata* (HBU), *Bungarus candidus* (BCA), *Python bivittatus* (PBI), *Naja kaouthia* (NKA), *Xenopeltis unicolor* (XUN), *Naja siamensis* (NSI), *Ptyas mucosa* (PMU), *Python regius* (PRE), *Acrochordus javanicus* (AJA). Light blue indicates male. Light red indicates female.

Quantification of Bov-B LINE copy number revealed differences among snake species when assessed using absolute quantification methods (Kruskal–Wallis rank sum test, *p* < 0.001) (Figure 2). The nuclear DNA content of *P. bivittatus* was reported by Castoe et al. [26]; the genome size is approximately 1.44 Gbp (including gaps). Quantification revealed that at least 9% × 10^−6^ to 5.63% × 10^−2^ (approximately 1682.34 copies per haploid genome) of the snake haploid genome was composed of Bov-B LINE sequences. *A. javanicus* showed the highest proportion (5.63% × 10^−2^ as 1814.70 copies) of Bov-B LINE in the genome, and *C. ruffus* exhibited the lowest proportion (9% × 10^−6^ as 2.77 × 10^−1^ copies) in the genome. 

### 3.5. Comparison of Bov-B LINE Copy Numbers between Males and Females

**Figure 3:**
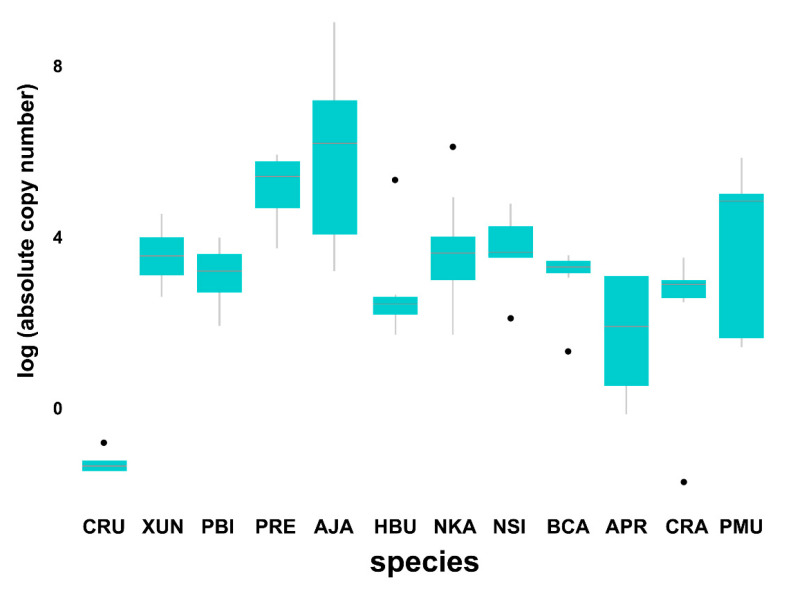
Boxplot showing accumulated copy number of *Bov-B* LINE retrotransposons between sexes in snake lineages determined by quantitative real-time PCR. *Cylindrophis ruffus* (CRU), *Xenopeltis unicolor* (XUN), *Python bivittatus* (PBI), *Python regius* (PRE), *Acrochordus javanicus* (AJA), *Homalopsis buccata* (HBU), *Naja kaouthia* (NKA), *Naja siamensis* (NSI), *Bungarus candidus* (BCA), *Ahaetulla prasina* (APR), *Coelognathus radiatus* (CRA), *Ptyas mucosa* (PMU).

The qPCR method was applied to measure the Bov-B LINE copy number between male and female individuals. Although the Wilcoxon signed-rank test showed no differentiation between sexes in all species (Wilcoxon signed-rank test, *W* = 687.5, *p* = 0.08) (Figure 3), males tended to show a higher copy number than females in five species (*X. unicolor*, *P. bivittatus*, *H. buccata*, *A. prasina*, and *P. mucosa*). By contrast, females tended to show a higher copy number than males in four species (*C. ruffus*, *P. regius*, *A. javanicus*, and *C. radiatus*). 

### 3.6. Divergence Time and Mutation Rate

The timing of phylogenetic divergence was estimated from sequence datasets for the mitochondrial cytochrome oxidase subunit 1 (*COI*) gene, nuclear-functional brain-derived neurotrophic factor (*BDNF*) gene, and Bov-B LINE independently (Figures S2 and S3). Divergence of henophidian and caenophidian snakes based on *COI* sequences was estimated to have occurred approximately 90 million years ago (95% highest posterior density interval: 84.31–95.40) during the Upper Cretaceous, and that based on *BDNF* sequences was approximately 90.86 MYA (95% HPD interval: 84.76–96.25) during the Upper Cretaceous. The Bov-B LINE groups I and II were estimated to have diverged from each other approximately 89.15 MYA (95% HPD interval 79.19–98.8) during the Upper Cretaceous. The substitution rates for *COI*, *BDNF*, and Bov-B LINE were 3.09 × 10^−3^ (95% HPD interval: 0.002635–0.003558), 2.40 × 10^−4^ (95% HPD interval: 0.00016–0.00037), and 1.37 × 10^−3^ (95% HPD interval: 0.001144–0.001642), respectively. 

## 4. Discussion

Bov-B LINE fragments in the portion of RT domain showed high similarity between distantly related species (mean similarity 84.68% ± 0.01) and similarities of 88% ± 0.01 for subgroup IA, 69% ± 0.01 for subgroup IB, and 90%± 0.01 for group II. This result was consistent with the patchy distribution of Bov-B LINE among vertebrates [17]. Inconsistencies between Bov-B LINE, *COI*, and *BDNF* tree topologies with snake phylogenies derived from other sequence datasets were also noted (Figures S2 and S3), which points to the incidence of HT events [17,19,80,81,82]. Furthermore, the emergence of Bov-B LINE independently in snake lineages is suggested with large different sequence similarity between group. Although mutation rates of Bov-B LINE were indicated to be more rapid than those of *COI* and *BDNF* in snake lineages, Bov-B LINEs have evolved at a slow rate compared with other TEs [9,83,84,85,86,87]. Average ratios of nonsynonymous to synonymous substitutions were generally less than one, which indicates that the RT genes evolved under purifying selection. This strongly suggests that Bov-B LINEs have been active during the evolution of snakes. 

### 4.1. Evolutionary History of BOV-B LINE in Snakes

Different HT events tend to show low degrees of similarity because mutations accumulate over time [44,88,89]. Similarity of more than 80% among TE sequences is indicative of recent HT events between species [17,19]. The Bov-B LINE from the domestic silkmoth (*Bombyx mori*) showed more than 80% similarity with the Bov-B LINE from *C. ruffus* and *Coelognathus flavolineatus* to bed bug (*Cimex lectularus*), which suggests that HTs have recently occurred between snakes and insects (Figure 1). Moreover, sequence similarity of 75% to 80% was observed between members of groups I and II, such as between *C. ruffus* and *Bungarus flaviceps* or between *X. unicolor* and *N. kaouthia*, which represent the majority of henophidian and caenophidian snakes, respectively. Interestingly, Bov-B LINE sequences of *V. salvator* were placed in subgroup IB and group II, and Bov-B LINE sequences of *E. maurus*, *C. horridus*, *H. buccata*, and *N. kaouthia* were placed in subgroup IA and group II (Figure 1). The presence of widely divergent sequences of Bov-B LINE in the same genome may also be considered to be indirect evidence of horizontal gene transfer. Such snails and slugs are the intermediate host between *Angiostrongylus cantonensis* and *A. costaricensis* and *Sorex araneus*, the common shrew [90,91]. To consider whether various individual TE copies appeared during independent HT events, pairwise divergence must be calculated between all individual TE copies and an ancestral founder copy. Pairwise divergence can be approximated from the consensus sequence of the Bov-B LINE, reconstructed using multiple copies belonging to the same TE family from a given species. Nucleotide sequences of each TE clade differed from each other by less than 20%, which is indicative of recent independent HT invasions. Generally, a difference in the estimated date of TE amplification–insertion between hosts may indicate that the TE was directionally transferred among snakes. In the present study with the specific location of Bov-B LINE sequences, the timing of the HT events can be approximated by estimating the divergence time and the pairwise divergence between all individual TE copies that show invasion times ranging from 79.19 to 98.8 MYA. This suggests that multiple HTs of Bov-B LINE in snakes, especially of henophidian and caenophidian snakes, occurred more recently within the same evolutionary timeframe. A possible ecological connection (e.g., parasitization or feeding) exists between insects and snakes [44]. Subgroup IB includes the majority of Bov-B LINE copies isolated from henophidian snakes and also contains insects (*Aedes aegypti*, *B. mori*, *Centruroides exilicauda*, *Danaus plexippus*, *Heliconius melpomene*, *Locusta migratoria*, and *Solenopsis invicta*), one of the bug’s (*Aedes aegypti*) preferred hosts in nature found in tropical and semitropical regions of the world [92,93]. A similar finding was noted for reptile ticks (*Amblyomma limbatum*), which are highly important parasites of domestic animals [94]. The tick species was placed with cenophidian snakes and monitor lizards in group II of the Bov-B LINE phylogeny, and the highest sequence similarity between the tick and any other species in group II was 90.9%. This finding suggests that host–parasite interactions might have facilitated HTs through frequent physical contact, such as between the saliva of the bug and the blood of its hosts whilst the bug is feeding. 

In the present study, we analyzed only a small number of Bov-B LINE copies for each species and a relatively small fragment (<400 bp) of the coding sequence. Further sampling of additional Bov-B LINE sequences and species, including blood-sucking and migratory insects capable of parasitizing snakes, is required for more precise estimation of the time and the geographical span of HTs. Identification of HT events between similar species or individuals of the same species using a genome-wide analysis will also provide the best option with a better understanding of the frequency of TE transfer, although this is complicated by the noise of vertically inherited and degraded TE copies. 

### 4.2. Independent Copy Number Variation among Snake Species

Diversification of Bov-B LINEs in snakes has resulted in the accumulation of large numbers of elements in a lineage-specific fashion ranging from 9 × 10^−6^% in *C. ruffus* to 5.63 × 10^−2^% in *A. javanicus* per haploid genome, as revealed by qPCR analysis. We also determined the proportion of each superfamily of Bov-B LINEs in the genome assemblies of each species. The same species showed substantial difference in estimated copy number between qPCR and the global estimation of TEs from the genome assemblies of *N. kaouthia* and *P. bivittatus*. This suggests that overlap of Bov-B LINE sequences might have been detected during genome assembly as the noise from high-throughput sequencing technologies, leading to the error of copy number estimation. The proportion of Bov-B LINEs is variable among species but is not directly associated with genome size or invasion time. By contrast, this reflects species specificity of TE invasion. Lower or higher abundances of Bov-B LINEs in a specific species might also be a consequence of genetic drift. However, the noise of random biological variation/association, especially with relatively small sample sizes could not be ruled out. Additionally, a full-length Bov-B LINE sequence is over 3kb in length [49]. Using a ranging of 330–385 bp RT domain to estimate Bov-B LINE copy number will likely retrieve most of the full-length copies present in the genome but may miss fragments or Bov-B LINE copies with a mutated RT domain region [17,95]. Updated genome annotation and more snake specimens are required to test this hypothesis. 

### 4.3. Bov-B LINE Copy Number Variation in Males and Females

The localization of TEs on snake sex chromosomes enables us to predict specific ratios of abundance between male and female individuals [35,36]. We addressed the question of whether the higher number of distinct Bov-B LINEs in snake genomes correlates with the presence of sex chromosome differentiation, representing a specific genomic context. To compare genomes of male and female individuals with the specific amplification of Bov-B LINEs on the Z and/or W sex chromosomes, we conducted a statistical analysis using the numbers of male versus female individuals for 12 snake species, which mostly exhibit heteromorphic sex chromosomes [36,96,97]. No snakes showed significant differences in Bov-B LINE copy number between males and females, possibly as a result of the low number of sample sizes examined. Moreover, many TEs show different activation in relation to age in several organisms (mammals and reptiles). This might result from different rates of DNA loss and insertion, and internal physiology with involving life span [98]. In addition, only a small proportion (0.32%) of the global total number of snake species were examined in this study, thus data for additional species and individuals are required to enable more substantive conclusions to be drawn. 

## 5. Conclusions

A snake phylogenetic framework with patchy distribution of Bov-B LINEs enables exploration of multiple HT events and the determination of the family to which they belong. This will allow additional, detailed analyses of the evolution of specific Bov-B LINE families in different species. A high degree of sequence conservation exists between snake lineages and other vertebrates, which is indicative of the evolutionary impact of purifying selection. Rapid speciation following the divergence of snakes approximately 150–170 MYA [99], coinciding with Bov-B LINE invasion into snake genomes, might lead to further hypotheses on the effect of Bov-B LINEs on genome structure and function, including the regulatory effects on transcriptional networks. This offers new prospects for research on the mechanisms of genomic and functional diversity. Increased annotation of available snake genome assemblies and improved systematic screening of snakes, other squamate reptiles, and their parasites may enhance our understanding of taxonomic and geographic spread of Bov-B LINEs, and lead to a greater appreciation of the impact of HT on the evolution of eukaryotic genomes. However, additional phylogenetic analyses are required based on portions of TEs other than the RT domain.

## Figures and Tables

**Table 1:** Classification of species and numbers of samples used in this study.

Species	Abbreviation	Superfamily	Family	Number of Individuals Used (Male + Female)
*Cylindrophis ruffus*	CRU	Henophidia	Cylindrophiidae	2 + 2
*Epicrates maurus*	EMA	Henophidia	Boidae	1 + 1
*Xenopeltis unicolor*	XUN	Henophidia	Xenopeltidae	3 + 3
*Python bivittatus*	PBI	Henophidia	Pythonidae	2 + 2
*Python regius*	PRE	Henophidia	Pythonidae	2 + 2
*Acrochordus javanicus*	AJA	Caenophidia	Acrochordidae	3 + 3
*Daboia siamensis*	DSI	Caenophidia	Viperidae	4 + 2
*Homalopsis buccata*	HBU	Caenophidia	Homalopsidae	3 + 2
*Enhydris enhydris*	EEN	Caenophidia	Homalopsidae	1 + 1
*Ophiophagus hannah*	OHA	Caenophidia	Elapidae	1 + 1
*Naja kaouthia*	NKA	Caenophidia	Elapidae	3 + 3
*Naja siamensis*	NSI	Caenophidia	Elapidae	3 + 2
*Bungarus candidus*	BCA	Caenophidia	Elapidae	4 + 4
*Bungarus flaviceps*	BFL	Caenophidia	Elapidae	1 + 1
*Oligodon fasciolatus*	OFA	Caenophidia	Colubridae	1 + 1
*Ahaetulla prasina*	APR	Caenophidia	Colubridae	2 + 2
*Boiga dendrophila*	BDE	Caenophidia	Colubridae	1 + 1
*Gonyosoma oxycephalum*	GOX	Caenophidia	Colubridae	1 + 1
*Coelognathus flavolineatus*	CFL	Caenophidia	Colubridae	1 + 1
*Coelognathus radiatus*	CRA	Caenophidia	Colubridae	2 + 4
*Xenochrophis flavipunctatus*	XFL	Caenophidia	Colubridae	1 + 1
*Ptyas mucosa*	PMU	Caenophidia	Colubridae	2 + 3
*Pantherophis guttatus*	PGU	Caenophidia	Colubridae	1 + 1
*Varanus salvator*	VSA	-	Varanidae	1 + 1
*Leiolepis reevesii*	LRE	-	Agamidae	1 + 1
*Gallus gallus*	GGA	-	Phasianidae	1 + 1

**Table 2:** Percentage of Bovine-B long interspersed nuclear element (Bov-B LINE) retrotransposon in the snake genome determined using BLASTN.

Species	Family	Accession No.	Percentage of *Bov-B* in Genome
*Python bivittatus*	Pythonidae	AEQU00000000.2	0.00115
*Protobothrops flavoviridis*	Viperidae	BFFQ00000000.1	0.01406
*Crotalus Pyrrhus*	Viperidae	JPMF00000000.1	0.00003
*Vipera berus beru*	Viperidae	JTGP00000000.1	0.01983
*Crotalus horridus*	Viperidae	LVCR00000000.1	0.00137
*Ophiophagus hannah*	Elapidae	AZIM00000000.1	0.00307
*Naja kaouthia*	Elapidae	PRJNA506318	0.00276
*Pantherophis guttatus*	Colubridae	JTLQ00000000.1	0.00121
*Thamnophis sirtalis*	Colubridae	LFLD00000000.1	0.00093

**Table 3:** Summary of nucleotide diversity (π) for each species used in this study.

Species	Length (bp)	*n*	%AT	Nucleotide Diversity (π) *	Accession Number
*Cylindrophis ruffus*	375–379	6	56.04	0.07 ± 0.02	LC365540-LC365545
*Epicrates maurus*	332–380	10	56.36	0.10 ± 0.02	LC365530-LC365539
*Xenopeltis unicolor*	375–381	8	59.26	0.10 ± 0.01	LC365508-LC365515
*Python bivittatus*	377–379	7	57.25	0.04 ± 0.00	LC365523-LC365529
*Python regius*	379	7	56.84	0.04 ± 0.01	LC365516-LC365522
*Acrochordus javanicus*	375–379	7	58.78	0.07 ± 0.01	LC365546-LC365552
*Daboia siamensis*	338–379	6	59.58	0.08 ± 0.01	LC365592-LC365597
*Homalopsis buccata*	364–379	5	59.57	0.13 ± 0.01	LC365610-LC365614
*Enhydris enhydris*	369–380	6	60.65	0.12 ± 0.01	LC365598-LC365603
*Ophiophagus hannah*	369–380	4	59.59	0.08 ± 0.01	LC365640-LC365643
*Naja kaouthia*	371–380	5	59.91	0.07 ± 0.01	LC365617-LC365621
*Naja siamensis*	345–379	9	59.96	0.12 ± 0.01	LC365623-LC365631
*Bungarus candidus*	342–379	5	59.96	0.11 ± 0.01	LC365559-LC365563
*Bungarus flaviceps*	369–377	6	60.79	0.14 ± 0.01	LC365633-LC365638
*Oligodon fasciolatus*	366–385	6	60.91	0.11 ± 0.01	LC365574-LC365579
*Ahaetulla prasina*	348–379	5	59.66	0.07 ± 0.01	LC365554-LC365558
*Boiga dendrophila*	366–381	8	59.73	0.10 ± 0.01	LC365565-LC365572
*Gonyosoma oxycephalum*	363–379	6	60.91	0.10 ± 0.01	LC365604-LC365609
*Coelognathus flavolineatus*	370–381	6	59.33	0.11 ± 0.01	LC365586-LC365590
*Coelognathus radiatus*	369–379	6	59.56	0.09 ± 0.01	LC384853-LC384858
*Xenochrophis flavipunctatus*	366–381	5	60.96	0.13 ± 0.02	LC365651-LC365655
*Ptyas mucosa*	378–379	4	59.75	0.10 ± 0.01	LC365645-LC365648
*Pantherophis guttatus*	359–385	8	59.52	0.08 ± 0.01	LC384860-LC384867
*Varanus salvator*	362–379	7	57.49	0.15 ± 0.02	LC365672-LC365678
*Leiolepis reevesii*	379	7	55.72	0.07 ± 0.01	LC365663-LC365669
*Gallus gallus*	372–379	6	55.98	0.04 ± 0.01	LC365657-LC365662

* Nucleotide diversity (π) ± SD of each snake species.

**Table 4:** Nucleotide diversity (π) of Bov-B LINE retrotransposon groups with outgroups excluded.

Group	*n*	Nucleotide Diversity (π)
**I**	55	0.11 ± 0.01
**II**	153	0.09 ± 0.00
**IA**	49	0.10 ± 0.01
**IB**	6	0.07 ± 0.02

**Table 5:** Nucleotide sequence divergence (*p*-distance) between Bov-B LINE retrotransposon groups with outgroups excluded.

Group	*p*-Distance
**I vs. II**	0.09 ± 0.01
**IA vs. IB**	0.09 ± 0.02

**Table 6:** Nucleotide diversity (π) of Bov-B LINE retrotransposon groups with outgroups included.

Group	*n*	Nucleotide Diversity (π)
**I**	124	0.22 ± 0.02
**II**	158	0.09 ± 0.00

**Table 7:** Nucleotide sequence divergence (*p*-distance) between Bov-B retrotransposon groups with outgroup included.

Group	*p*-Distance
**I vs. II**	0.18 ± 0.04
**IA vs. IB**	0.24 ± 0.04
**IA vs. II**	0.12 ± 0.04
**IB vs. II**	0.24 ± 0.04

## Supplementary Materials

The following are available online at https://www.mdpi.com/2073-4425/11/11/1241/s1, Table S1. Summary of BDNF sequence of each species used in this study, Table S2. Summary of COI sequence of each species used in this study, Table S3. Phylogenetic groups of Bov-B LINE retrotransposon sequences among eight snake families and outgroups inferred using maximum likelihood analysis, Table S4. Pairwise comparison of nucleotide sequence divergence (*p*-distance) of Bov-B LINE retrotransposon among 32 snake species, Table S5. Nonsynonymous substitution sites (*K*_a_) per synonymous substitution site (*K*_s_) of Bov-B LINE retrotransposon among 32 snake species, Figure S1. Standard curve for Bov-B LINE copies amplified by specific primers. The DNA dilution series ranged from 0.25 to 25.00 ng/µl of genomic DNA from radiated ratsnake (*Coelognathus radiatus*) (*n*= 5). The *x*-axis is the log-concentration of genomic DNA, and the *y*-axis is the cycle threshold value. Regression line calculated as *R*^2^ = 0.9627, *p* < 0.01, Figure S2. Bayesian phylognetic tree of *COI* gene. The number at each node indicates the estimated divergence time. The numbers in black circles indicate four calibration points that were used for the evolutionary rate estimation: 1) 91.0 million years ago (MYA) for the time to the most recent common ancestor (TMRCA) of all sequences, 2) 45 MYA for the TMRCA of Colubridae, 3) 35 MYA for the TMRCA of Elapidae, and 4) 24 MYA for the divergence time between *Enhydris* and *Homalopsis*, Figure S3. Bayesian phylognetic tree of *BDNF* gene. The number at each node indicates the estimated divergence time. The numbers in black circles indicate four calibration points that were used for the evolutionary rate estimation: 1) 91.0 million years ago (MYA) for the time to the most recent common ancestor (TMRCA) of all sequences, 2) 45 MYA for the TMRCA of Colubridae, 3) 35 MYA for the TMRCA of Elapidae, and 4) 24 MYA for the divergence time between *Enhydris* and *Homalopsis*.
