# Supplementary material for: Existence of Bov-B LINE Retrotransposons in Snake Lineages Reveals Recent Multiple Horizontal Gene Transfers with Copy Number Variation
Source: Genes (Basel). 2020 Oct 22;11(11):1241. doi: 10.3390/genes11111241 (PMC7716205; doi:10.3390/genes11111241)
Supplement: Supplementary file 1 [file genes-11-01241-s001.zip › supplementary table/Table S1.docx]

**Table S1.** Summary of *BDNF* sequence of each species used in this study.

| No. | BDNF sequence name | Species | Families | Abbreviation | Code | Accession no. |
| --- | --- | --- | --- | --- | --- | --- |
| 1 | Acrochordus javanicus AJ01 | *Acrochordus javanicus* | Acrochordidae | AJA | AJ01 | LC519929 |
| 2 | Ahaetulla prasina AP01 | *Ahaetulla prasina* | Colubridae | APR | AP01 | LC519930 |
| 3 | Ahaetulla prasina AP02 | *Ahaetulla prasina* | Colubridae | APR | AP02 | LC519931 |
| 4 | Ahaetulla prasina AP03 | *Ahaetulla prasina* | Colubridae | APR | AP03 | LC519932 |
| 5 | Ahaetulla prasina AP04 | *Ahaetulla prasina* | Colubridae | APR | AP04 | LC519933 |
| 6 | Boiga dendrophila BD01 | *Boiga dendrophila* | Colubridae | BDE | BD01 | LC519934 |
| 7 | Boiga dendrophila BD02 | *Boiga dendrophila* | Colubridae | BDE | BD02 | LC519935 |
| 8 | Boiga dendrophila BD03 | *Boiga dendrophila* | Colubridae | BDE | BD03 | LC519936 |
| 9 | Bungarus candidus BC01 | *Bungarus candidus* | Elapidae | BCA | BCA1 | LC519937 |
| 10 | Bungarus candidus BC03 | *Bungarus candidus* | Elapidae | BCA | BCA2 | LC519938 |
| 11 | Bungarus flaviceps BFLA01 | *Bungarus flaviceps* | Elapidae | BFL | BFLA01 | LC519939 |
| 12 | Bungarus flaviceps BFLA02 | *Bungarus flaviceps* | Elapidae | BFL | BFLA02 | LC519940 |
| 13 | Coelognathus flavolineathus CF01f | *Coelognathus flavolineathus* | Colubridae | CFL | CF01f | LC519941 |
| 14 | Coelognathus flavolineathus CF01m | *Coelognathus flavolineathus* | Colubridae | CFL | CF01m | LC519942 |
| 15 | Coelognathus radiatus CRA02 | *Coelognathus radiatus* | Colubridae | CRA | ER02/CRA01 | LC519943 |
| 16 | Coelognathus radiatus CRA03 | *Coelognathus radiatus* | Colubridae | CRA | ER03/CRA02 | LC519944 |
| 17 | Coelognathus radiatus CRA04 | *Coelognathus radiatus* | Colubridae | CRA | ER04/CRA03 | LC519945 |
| 18 | Cylindrophis ruffus CRU | *Cylindrophis ruffus* | Cylindrophiidae | CRU | CRU02 | LC519946 |
| 19 | Daboia siamensis DSI | *Daboia siamensis* | Viperidae | DSI | DRUm | LC519947 |
| 20 | Epicrates maurus EMA | *Epicrates maurus* | Boidae | EMA | EMAbdnf | LC533895 |
| 21 | Enhydris enhydris EE01 | *Enhydris enhydris* | Homalopsidae | EEN | EE01 | LC519948 |
| 22 | Enhydris enhydris EE02 | *Enhydris enhydris* | Homalopsidae | EEN | EE02 | LC519949 |
| 23 | Enhydris enhydris EE03 | *Enhydris enhydris* | Homalopsidae | EEN | EE03 | LC519950 |
| 24 | Enhydris enhydris EE04 | *Enhydris enhydris* | Homalopsidae | EEN | EE04 | LC519951 |
| 25 | Gonyosoma oxycephalum GOX | *Gonyosoma oxycephalum* | Colubridae | GOX | GOXbdnf | LC533896 |
| 26 | Homalopsis buccata HB01 | *Homalopsis buccata* | Homalopsidae | HBU | HB01 | LC519952 |
| 27 | Homalopsis buccata HB02 | *Homalopsis buccata* | Homalopsidae | HBU | HB02 | LC519953 |
| 28 | Homalopsis buccata HB03 | *Homalopsis buccata* | Homalopsidae | HBU | HB03 | LC519954 |
| 29 | Homalopsis buccata HB04 | *Homalopsis buccata* | Homalopsidae | HBU | HB04 | LC519955 |
| 30 | Naja siamensis NSI01f | *Naja siamensis* | Elapidae | NSI | SC01f | LC519956 |
| 31 | Naja siamensis NSI01m | *Naja siamensis* | Elapidae | NSI | SC01m | LC519957 |
| 32 | Naja siamensis NSI02f | *Naja siamensis* | Elapidae | NSI | SC02f | LC519958 |
| 33 | Naja siamensis NSI03m | *Naja siamensis* | Elapidae | NSI | SC03m | LC519959 |
| 34 | Naja kaouthia NKA | *Naja kaouthia* | Elapidae | NKA | NKAbdnf | LC533897 |
| 35 | Oligodon fasciolatus OFA | *Oligodon fasciolatus* | Colubridae | OFA | OFAbdnf | LC533898 |
| 36 | Ophiophagus hannah OHA2 | *Ophiophagus hannah* | Elapidae | OHA | OH01 | LC519960 |
| 37 | Ophiophagus hannah OHA1 | *Ophiophagus hannah* | Elapidae | OHA | OHAf | LC519961 |
| 38 | Ophiophagus hannah OHA3 | *Ophiophagus hannah* | Elapidae | OHA | OHAm | LC519962 |
| 39 | Pantherophis guttatus PGUf | *Pantherophis guttatus* | Colubridae | PGU | PGUf | LC519963 |
| 40 | Pantherophis guttatus PGUm | *Pantherophis guttatus* | Colubridae | PGU | PGUm | LC519964 |
| 41 | Python regius PRE | *Python regius* | Pythonidae | PRE | PREbdnf | LC533899 |
| 42 | Ptyas mucosus PM01 | *Ptyas mucosus* | Colubridae | PMU | PM01 | LC519965 |
| 43 | Ptyas mucosus PM02 | *Ptyas mucosus* | Colubridae | PMU | PM02 | LC519966 |
| 44 | Ptyas mucosus PM03 | *Ptyas mucosus* | Colubridae | PMU | PM03 | LC519967 |
| 45 | Ptyas mucosus PM04 | *Ptyas mucosus* | Colubridae | PMU | PM04 | LC519968 |
| 46 | Python bivittatus PB01 | *Python bivittatus* | Pythonidae | PBI | PB01 | LC519969 |
| 47 | Python bivittatus PB04 | *Python bivittatus* | Pythonidae | PBI | PB04 | LC519970 |
| 48 | Xenochrophis flavipunctatus XF01 | *Xenochrophis flavipunctatus* | Colubridae | XFL | XF01 | LC519971 |
| 49 | Xenochrophis flavipunctatus XF02 | *Xenochrophis flavipunctatus* | Colubridae | XFL | XF02 | LC519972 |
| 50 | Xenochrophis flavipunctatus XF03 | *Xenochrophis flavipunctatus* | Colubridae | XFL | XF03 | LC519973 |
| 51 | Xenopeltis unicolor XU01m | *Xenopeltis unicolor* | Xenopeltidae | XUN | XU01f | LC519974 |
| 52 | Xenopeltis unicolor XU02m | *Xenopeltis unicolor* | Xenopeltidae | XUN | XU01m | LC519975 |
| 53 | Xenopeltis unicolor XU02f | *Xenopeltis unicolor* | Xenopeltidae | XUN | XU02f | LC519976 |
| 54 | Xenopeltis unicolor XU01f | *Xenopeltis unicolor* | Xenopeltidae | XUN | XU02m | LC519977 |
