# Supplementary material for: Existence of Bov-B LINE Retrotransposons in Snake Lineages Reveals Recent Multiple Horizontal Gene Transfers with Copy Number Variation
Source: Genes (Basel). 2020 Oct 22;11(11):1241. doi: 10.3390/genes11111241 (PMC7716205; doi:10.3390/genes11111241)
Supplement: Supplementary file 1 [file genes-11-01241-s001.zip › supplementary table/Table S2.docx]

**Table S2.** Summary of *COI* sequence of each species used in this study.

| No. | *COI* sequence name | Species | Families | Abbreviation | Code | Accession no. |
| --- | --- | --- | --- | --- | --- | --- |
| 1 | A_javanicus_AJA_COI | *Acrochordus javanicus* | Acrochordidae | AJA | AJACOI | LC533890 |
| 2 | A_prasina_APR | *Ahaetulla prasina* | Colubridae | APR | APR | LC075335 |
| 3 | A_prasina_APR01 | *Ahaetulla prasina* | Colubridae | APR | APR01 | LC075334 |
| 4 | B_dendrophil_BDE02 | *Boiga dendrophila* | Colubridae | BDE | BDE02 | LC075336 |
| 5 | B_dendrophil_BDE04 | *Boiga dendrophila* | Colubridae | BDE | BDE04 | LC075337 |
| 6 | Boiga_dendrophila_BD01 | *Boiga dendrophila* | Colubridae | BDE | BDE01 | LC519978 |
| 7 | Boiga_dendrophila_BD02 | *Boiga dendrophila* | Colubridae | BDE | BDE02 | LC519979 |
| 8 | B_candidus_BCA1 | *Bungarus candidus* | Elapidae | BCA | BCA1 | AB920192 |
| 9 | B_candidus_BCA2 | *Bungarus candidus* | Elapidae | BCA | BCA2 | AB920193 |
| 10 | B_candidus_BCA3 | *Bungarus candidus* | Elapidae | BCA | BCA3 | AB920194 |
| 11 | Bungarus_candidus_BC01 | *Bungarus candidus* | Elapidae | BCA | BCA01 | LC519980 |
| 12 | Bungarus_candidus_BC03 | *Bungarus candidus* | Elapidae | BCA | BCA03 | LC519981 |
| 13 | C_flavolineatus_CFL01 | *Coelognathus flavolineathus* | Colubridae | CFL | CFL01 | LC075340 |
| 14 | C_flavolineatus_CFL02 | *Coelognathus flavolineathus* | Colubridae | CFL | CFL02 | LC075341 |
| 15 | C_radiatus_ER01 | *Coelognathus radiatus* | Colubridae | CRA | CRA01 | LC105609 |
| 16 | C_radiatus_ER02 | *Coelognathus radiatus* | Colubridae | CRA | CRA02 | LC105610 |
| 17 | C_radiatus_ER03 | *Coelognathus radiatus* | Colubridae | CRA | CRA03 | LC105611 |
| 18 | C_radiatus_ER04 | *Coelognathus radiatus* | Colubridae | CRA | CRA04 | LC105612 |
| 19 | C_ruffus_CRU01m | *Cylindrophis ruffus* | Cylindrophiidae | CRU | CRU01m | LC075327 |
| 20 | C_ruffus_CRU02f | *Cylindrophis ruffus* | Cylindrophiidae | CRU | CRU02f | LC075328 |
| 21 | D_siamensis_DRU1 | *Daboia siamensis* | Viperidae | DSI | DSI1 | AB920195 |
| 22 | D_siamensis_DRU2 | *Daboia siamensis* | Viperidae | DSI | DSI2 | AB920196 |
| 23 | D_siamensis_DRU4 | *Daboia siamensis* | Viperidae | DSI | DSI4 | LC533894 |
| 24 | E_enhydris_EE01 | *Enhydris enhydris* | Homalopsidae | EEN | EEN01 | LC105601 |
| 25 | E_enhydris_EE02 | *Enhydris enhydris* | Homalopsidae | EEN | EEN02 | LC105602 |
| 26 | E_enhydris_EE03 | *Enhydris enhydris* | Homalopsidae | EEN | EEN03 | LC105603 |
| 27 | E_enhydris_EE04 | *Enhydris enhydris* | Homalopsidae | EEN | EEN04 | LC105604 |
| 28 | E_maurus_EMA_COI | *Epicrates maurus* | Boidae | EMA | EMACOI | LC533888 |
| 29 | G_oxycephalum_GOX01 | *Gonyosoma oxycephalum* | Colubridae | GOX | GOX01 | LC075338 |
| 30 | G_oxycephalum_GOX02 | *Gonyosoma oxycephalum* | Colubridae | GOX | GOX02 | LC075339 |
| 31 | H_buccata_HB02 | *Homalopsis buccata* | Homalopsidae | HBU | HBU02 | LC105599 |
| 32 | H_buccata_HB04 | *Homalopsis buccata* | Homalopsidae | HBU | HBU04 | LC105600 |
| 33 | H_buccata_HBU01 | *Homalopsis buccata* | Homalopsidae | HBU | HBU01 | LC075330 |
| 34 | H_buccata_HBU03 | *Homalopsis buccata* | Homalopsidae | HBU | HBU03 | LC075331 |
| 35 | N. kaouthia_NKA4 | *Naja kaouthia* | Elapidae | NNK | NNK4 | LC533893 |
| 36 | N_kaouthia_NKA1 | *Naja kaouthia* | Elapidae | NKA | NKA1 | AB920183 |
| 37 | N_kaouthia_NKA2 | *Naja kaouthia* | Elapidae | NKA | NKA2 | AB920184 |
| 38 | N_kaouthia_NKA3 | *Naja kaouthia* | Elapidae | NKA | NKA3 | AB920185 |
| 39 | N_siamensis_NSI2 | *Naja siamensis* | Elapidae | NSI | NSI2 | AB920187 |
| 40 | N_siamensis_NSI3 | *Naja siamensis* | Elapidae | NSI | NSI3 | AB920188 |
| 41 | N_siamensis_SC01f | *Naja siamensis* | Elapidae | NSI | NSI01f | LC086063 |
| 42 | O_fasciolatus_OF02 | *Oligodon fasciolatus* | Colubridae | OFA | OFA02 | LC105613 |
| 43 | O_fasciolatus_OFA01 | *Oligodon fasciolatus* | Colubridae | OFA | OFA01 | LC075332 |
| 44 | O_fasciolatus_OFA03 | *Oligodon fasciolatus* | Colubridae | OFA | OFA03 | LC075333 |
| 45 | O_hannah_OHA1 | *Ophiophagus hannah* | Elapidae | OHA | OHA1 | AB920180 |
| 46 | O_hannah_OHA2 | *Ophiophagus hannah* | Elapidae | OHA | OHA2 | AB920181 |
| 47 | O_hannah_OHA3 | *Ophiophagus hannah* | Elapidae | OHA | OHA3 | AB920182 |
| 48 | O_hannah_OHA4 | *Ophiophagus hannah* | Elapidae | OHA | OHA4 | LC533892 |
| 49 | Ophiophagus_hannah_OH01 | *Ophiophagus hannah* | Elapidae | OHA | OHA01 | LC519982 |
| 50 | P_guttatus_PGU_COI | *Pantherophis guttatus* | Colubridae | PGU | PGUU_COI | LC533891 |
| 51 | P_mucosa_PM01 | *Ptyas mucosus* | Colubridae | PMU | PMU01 | LC105605 |
| 52 | P_mucosa_PM02 | *Ptyas mucosus* | Colubridae | PMU | PMU02 | LC105606 |
| 53 | P_mucosa_PM03 | *Ptyas mucosus* | Colubridae | PMU | PMU03 | LC105607 |
| 54 | P_mucosa_PM04 | *Ptyas mucosus* | Colubridae | PMU | PMU04 | LC105608 |
| 55 | P_bivittatus_PB01 | *Python bivittatus* | Pythonidae | PBI | PBI01 | LC086062 |
| 56 | P_bivittatus_PB04 | *Python bivittatus* | Pythonidae | PBI | PBI04 | LC075329 |
| 57 | P_regius_PRE_COI | *Python regius* | Pythonidae | PRE | PRECOI | LC533889 |
| 58 | X_flavipunctatus_XF02 | *Xenochrophis flavipunctatus* | Colubridae | XFL | XFL02 | LC105614 |
| 59 | X_flavipunctatus_XFL01 | *Xenochrophis flavipunctatus* | Colubridae | XFL | XFL01 | LC075343 |
| 60 | X_flavipunctatus_XFL03 | *Xenochrophis flavipunctatus* | Colubridae | XFL | XFL03 | LC075343 |
| 61 | X_unicolor_XUN01m | *Xenopeltis unicolor* | Xenopeltidae | XUN | XUN01m | LC074882 |
| 62 | X_unicolor_XUN02f | *Xenopeltis unicolor* | Xenopeltidae | XUN | XUN02f | LC075326 |
| 63 | Xenopeltis_unicolor_XU02 | *Xenopeltis unicolor* | Xenopeltidae | XUN | XUN02 | LC519983 |
