# Supplementary material for: Existence of Bov-B LINE Retrotransposons in Snake Lineages Reveals Recent Multiple Horizontal Gene Transfers with Copy Number Variation
Source: Genes (Basel). 2020 Oct 22;11(11):1241. doi: 10.3390/genes11111241 (PMC7716205; doi:10.3390/genes11111241)
Supplement: Supplementary file 1 [file genes-11-01241-s001.zip › supplementary table/Table S3.docx]

**Table S3.** Phylogenetic groups of Bov-B LINE retrotransposon sequences among eight snake families and outgroups inferred using maximum likelihood analysis.

| No. | Group | Sample code | Species | Family | Class |
| --- | --- | --- | --- | --- | --- |
| 1 | IA | AJAF001 | *Acrochordus javanicus* | Acrochordidae | Reptilia |
| 2 | IA | AJAF002 | *Acrochordus javanicus* | Acrochordidae | Reptilia |
| 3 | IA | AJAF003 | *Acrochordus javanicus* | Acrochordidae | Reptilia |
| 4 | IA | AJAF004 | *Acrochordus javanicus* | Acrochordidae | Reptilia |
| 5 | IA | AJAM001 | *Acrochordus javanicus* | Acrochordidae | Reptilia |
| 6 | IA | AJAM002 | *Acrochordus javanicus* | Acrochordidae | Reptilia |
| 7 | IA | AJAM003 | *Acrochordus javanicus* | Acrochordidae | Reptilia |
| 8 | IA | BCON001 | *Boa constrictor* | ‎Boidae | Reptilia |
| 9 | IA | CHON003 | *Crotalus horridus* | Viperidae | Reptilia |
| 10 | IA | CPYN001 | *Crotalus pyrrhus* | Viperidae | Reptilia |
| 11 | IA | HBUM003 | *Homalopsis buccata* | Homalopsidae | Reptilia |
| 12 | IA | NKAN002 | *Naja kaouthia* | Elapidae | Reptilia |
| 13 | IA | EMAF001 | *Epicrates maurus* | Boidae | Reptilia |
| 14 | IA | EMAF002 | *Epicrates maurus* | Boidae | Reptilia |
| 15 | IA | EMAF003 | *Epicrates maurus* | Boidae | Reptilia |
| 16 | IA | EMAM001 | *Epicrates maurus* | Boidae | Reptilia |
| 17 | IA | EMAM002 | *Epicrates maurus* | Boidae | Reptilia |
| 18 | IA | EMAM003 | *Epicrates maurus* | Boidae | Reptilia |
| 19 | IA | EMAM005 | *Epicrates maurus* | Boidae | Reptilia |
| 20 | IA | PBIN001 | *Python bivittatus* | Pythonidae | Reptilia |
| 21 | IA | PBIN002 | *Python bivittatus* | Pythonidae | Reptilia |
| 22 | IA | PBIN003 | *Python bivittatus* | Pythonidae | Reptilia |
| 23 | IA | PBIN004 | *Python bivittatus* | Pythonidae | Reptilia |
| 24 | IA | PBIN005 | *Python bivittatus* | Pythonidae | Reptilia |
| 25 | IA | PBIN006 | *Python bivittatus* | Pythonidae | Reptilia |
| 26 | IA | PBIF001 | *Python bivittatus* | Pythonidae | Reptilia |
| 27 | IA | PBIF002 | *Python bivittatus* | Pythonidae | Reptilia |
| 28 | IA | PBIF003 | *Python bivittatus* | Pythonidae | Reptilia |
| 29 | IA | PBIF004 | *Python bivittatus* | Pythonidae | Reptilia |
| 30 | IA | PBIM001 | *Python bivittatus* | Pythonidae | Reptilia |
| 31 | IA | PBIM002 | *Python bivittatus* | Pythonidae | Reptilia |
| 32 | IA | PBIM003 | *Python bivittatus* | Pythonidae | Reptilia |
| 33 | IA | PREF001 | *Python regius* | Pythonidae | Reptilia |
| 34 | IA | PREF002 | *Python regius* | Pythonidae | Reptilia |
| 35 | IA | PREF003 | *Python regius* | Pythonidae | Reptilia |
| 36 | IA | PREM001 | *Python regius* | Pythonidae | Reptilia |
| 37 | IA | PREM002 | *Python regius* | Pythonidae | Reptilia |
| 38 | IA | PREM003 | *Python regius* | Pythonidae | Reptilia |
| 39 | IA | PREM004 | *Python regius* | Pythonidae | Reptilia |
| 40 | IA | XUNF001 | *Xenopeltis unicolor* | Xenopeltidae | Reptilia |
| 41 | IA | XUNF002 | *Xenopeltis unicolor* | Xenopeltidae | Reptilia |
| 42 | IA | XUNF003 | *Xenopeltis unicolor* | Xenopeltidae | Reptilia |
| 43 | IA | XUNF004 | *Xenopeltis unicolor* | Xenopeltidae | Reptilia |
| 44 | IA | XUNF005 | *Xenopeltis unicolor* | Xenopeltidae | Reptilia |
| 45 | IA | XUNM001 | *Xenopeltis unicolor* | Xenopeltidae | Reptilia |
| 46 | IA | XUNM002 | *Xenopeltis unicolor* | Xenopeltidae | Reptilia |
| 47 | IA | XUNM003 | *Xenopeltis unicolor* | Xenopeltidae | Reptilia |
| 48 | IA | CATN001 | *Ctenotus atlas* | Scincidae | Reptilia |
| 49 | IA | ERIN001 | *Eremiascincus richardsonii* | Scincidae | Reptilia |
| 50 | IA | BMUN001 | *Bos mutus* | Bovidae | Mammalia |
| 51 | IA | BMUN002 | *Bos mutus* | Bovidae | Mammalia |
| 52 | IA | BMUN003 | *Bos mutus* | Bovidae | Mammalia |
| 53 | IA | BMUN004 | *Bos mutus* | Bovidae | Mammalia |
| 54 | IA | BTAN001 | *Bos taurus* | Bovidae | Mammalia |
| 55 | IA | BTAN002 | *Bos taurus* | Bovidae | Mammalia |
| 56 | IA | BTAN003 | *Bos taurus* | Bovidae | Mammalia |
| 57 | IA | BTAN004 | *Bos taurus* | Bovidae | Mammalia |
| 58 | IA | OARN001 | *Ovis aries* | Bovidae | Mammalia |
| 59 | IA | OCAN001 | *Ovis canadensis* | Bovidae | Mammalia |
| 60 | IA | OCAN002 | *Ovis canadensis* | Bovidae | Mammalia |
| 61 | IA | OCAN003 | *Ovis canadensis* | Bovidae | Mammalia |
| 62 | IA | OCAN004 | *Ovis canadensis* | Bovidae | Mammalia |
| 63 | IA | OCAN005 | *Ovis canadensis* | Bovidae | Mammalia |
| 64 | IA | OCAN006 | *Ovis canadensis* | Bovidae | Mammalia |
| 65 | IA | OCAN007 | *Ovis canadensis* | Bovidae | Mammalia |
| 66 | IA | OCAN008 | *Ovis canadensis* | Bovidae | Mammalia |
| 67 | IA | PBRN001 | *Petaurus breviceps* | Petauridae | Mammalia |
| 68 | IB | GGAF001 | *Gallus gallus* | Phasianidae | Aves |
| 69 | IB | GGAF002 | *Gallus gallus* | Phasianidae | Aves |
| 70 | IB | GGAF003 | *Gallus gallus* | Phasianidae | Aves |
| 71 | IB | GGAM001 | *Gallus gallus* | Phasianidae | Aves |
| 72 | IB | GGAM002 | *Gallus gallus* | Phasianidae | Aves |
| 73 | IB | GGAM003 | *Gallus gallus* | Phasianidae | Aves |
| 74 | IB | CRUF001 | *Cylindrophis ruffus* | Cylindrophiidae | Reptilia |
| 75 | IB | CRUF002 | *Cylindrophis ruffus* | Cylindrophiidae | Reptilia |
| 76 | IB | CRUF003 | *Cylindrophis ruffus* | Cylindrophiidae | Reptilia |
| 77 | IB | CRUM001 | *Cylindrophis ruffus* | Cylindrophiidae | Reptilia |
| 78 | IB | CRUM002 | *Cylindrophis ruffus* | Cylindrophiidae | Reptilia |
| 79 | IB | CRUM003 | *Cylindrophis ruffus* | Cylindrophiidae | Reptilia |
| 80 | IB | AAEN001 | *Aedes aegypti* | Culicidae | Insecta |
| 81 | IB | BMON001 | *Bombyx mori* | Bombycidae | Insecta |
| 82 | IB | BMON002 | *Bombyx mori* | Bombycidae | Insecta |
| 83 | IB | BMON003 | *Bombyx mori* | Bombycidae | Insecta |
| 84 | IB | BMON004 | *Bombyx mori* | Bombycidae | Insecta |
| 85 | IB | CEXN001 | *Centruroides exilicauda* | Buthidae | Arachnida |
| 86 | IB | DPLN001 | *Danaus plexippus* | Nymphalidae | Insecta |
| 87 | IB | DPLN002 | *Danaus plexippus* | Nymphalidae | Insecta |
| 88 | IB | DPLN003 | *Danaus plexippus* | Nymphalidae | Insecta |
| 89 | IB | DPLN004 | *Danaus plexippus* | Nymphalidae | Insecta |
| 90 | IB | HMEN001 | *Heliconius melpomene* | Nymphalidae | Insecta |
| 91 | IB | LMIN001 | *Locusta migratoria* | Acrididae | Insecta |
| 92 | IB | LMIN002 | *Locusta migratoria* | Acrididae | Insecta |
| 93 | IB | LMIN003 | *Locusta migratoria* | Acrididae | Insecta |
| 94 | IB | SINN001 | *Solenopsis invicta* | Formicidae | Insecta |
| 95 | IB | SINN002 | *Solenopsis invicta* | Formicidae | Insecta |
| 96 | IB | ACAN001 | *Anolis carolinensis* | Dactyloidae | Reptilia |
| 97 | IB | ACAN002 | *Anolis carolinensis* | Dactyloidae | Reptilia |
| 98 | IB | ANON001 | *Amphibolurus norrisi* | Agamidae | Reptilia |
| 99 | IB | DRAN001 | *Darevskia raddei* | Lacertidae | Reptilia |
| 100 | IB | DRAN002 | *Darevskia raddei* | Lacertidae | Reptilia |
| 101 | IB | DRAN003 | *Darevskia raddei* | Lacertidae | Reptilia |
| 102 | IB | DUNN001 | *Darevskia unisexualis* | Lacertidae | Reptilia |
| 103 | IB | DUNN002 | *Darevskia unisexualis* | Lacertidae | Reptilia |
| 104 | IB | DUNN003 | *Darevskia unisexualis* | Lacertidae | Reptilia |
| 105 | IB | DVAN001 | *Darevskia valentini* | Lacertidae | Reptilia |
| 106 | IB | DVAN002 | *Darevskia valentini* | Lacertidae | Reptilia |
| 107 | IB | DVAN003 | *Darevskia valentini* | Lacertidae | Reptilia |
| 108 | IB | GLAN001 | *Gehyra lazelli* | Gekkonidae | Reptilia |
| 109 | IB | GVAN001 | *Gehyra variegata* | Gekkonidae | Reptilia |
| 110 | IB | LREF001 | *Leiolepis reevesii* | Agamidae | Reptilia |
| 111 | IB | LREF002 | *Leiolepis reevesii* | Agamidae | Reptilia |
| 112 | IB | LREF003 | *Leiolepis reevesii* | Agamidae | Reptilia |
| 113 | IB | LREM001 | *Leiolepis reevesii* | Agamidae | Reptilia |
| 114 | IB | LREM002 | *Leiolepis reevesii* | Agamidae | Reptilia |
| 115 | IB | LREM003 | *Leiolepis reevesii* | Agamidae | Reptilia |
| 116 | IB | LREM004 | *Leiolepis reevesii* | Agamidae | Reptilia |
| 117 | IB | LREM005 | *Leiolepis reevesii* | Agamidae | Reptilia |
| 118 | IB | VSAF004 | *Varanus salvator* | Varanidae | Reptilia |
| 119 | IB | VSAM001 | *Varanus salvator* | Varanidae | Reptilia |
| 120 | IB | VSAM003 | *Varanus salvator* | Varanidae | Reptilia |
| 121 | IB | ECAN001 | *Equus ferus caballus* | Equidae | Mammalia |
| 122 | IB | PCAN001 | *Procavia capensis* | Procaviidae | Mammalia |
| 123 | IB | TACN001 | *Tachyglossus aculeatus* | Tachyglossidae | Mammalia |
| 124 | IB | DREN001 | *Danio rerio* | Cyprinidae | Actinopterygii |
| 125 | II | ACON001 | *Agkistrodon contortrix* | Viperidae | Reptilia |
| 126 | II | APRF001 | *Ahaetulla prasina* | Colubridae | Reptilia |
| 127 | II | APRF002 | *Ahaetulla prasina* | Colubridae | Reptilia |
| 128 | II | APRM001 | *Ahaetulla prasina* | Colubridae | Reptilia |
| 129 | II | APRM002 | *Ahaetulla prasina* | Colubridae | Reptilia |
| 130 | II | APRM003 | *Ahaetulla prasina* | Colubridae | Reptilia |
| 131 | II | BCAF001 | *Bungarus candidus* | Elapidae | Reptilia |
| 132 | II | BCAF002 | *Bungarus candidus* | Elapidae | Reptilia |
| 133 | II | BCAF003 | *Bungarus candidus* | Elapidae | Reptilia |
| 134 | II | BCAM001 | *Bungarus candidus* | Elapidae | Reptilia |
| 135 | II | BCAM002 | *Bungarus candidus* | Elapidae | Reptilia |
| 136 | II | BDEF001 | *Boiga dendrophila* | Colubridae | Reptilia |
| 137 | II | BDEF002 | *Boiga dendrophila* | Colubridae | Reptilia |
| 138 | II | BDEF003 | *Boiga dendrophila* | Colubridae | Reptilia |
| 139 | II | BDEM001 | *Boiga dendrophila* | Colubridae | Reptilia |
| 140 | II | BDEM002 | *Boiga dendrophila* | Colubridae | Reptilia |
| 141 | II | BDEM003 | *Boiga dendrophila* | Colubridae | Reptilia |
| 142 | II | BDEM004 | *Boiga dendrophila* | Colubridae | Reptilia |
| 143 | II | BDEM005 | *Boiga dendrophila* | Colubridae | Reptilia |
| 144 | II | BDEM006 | *Boiga dendrophila* | Colubridae | Reptilia |
| 145 | II | BFLF001 | *Bungarus flaviceps* | Elapidae | Reptilia |
| 146 | II | BFLF002 | *Bungarus flaviceps* | Elapidae | Reptilia |
| 147 | II | BFLF003 | *Bungarus flaviceps* | Elapidae | Reptilia |
| 148 | II | BFLM001 | *Bungarus flaviceps* | Elapidae | Reptilia |
| 149 | II | BFLM002 | *Bungarus flaviceps* | Elapidae | Reptilia |
| 150 | II | BFLM003 | *Bungarus flaviceps* | Elapidae | Reptilia |
| 151 | II | CFLF001 | *Coelognathus flavolineatus* | Colubridae | Reptilia |
| 152 | II | CFLF002 | *Coelognathus flavolineatus* | Colubridae | Reptilia |
| 153 | II | CFLF003 | *Coelognathus flavolineatus* | Colubridae | Reptilia |
| 154 | II | CFLM001 | *Coelognathus flavolineatus* | Colubridae | Reptilia |
| 155 | II | CFLM002 | *Coelognathus flavolineatus* | Colubridae | Reptilia |
| 156 | II | CFLM003 | *Coelognathus flavolineatus* | Colubridae | Reptilia |
| 157 | II | CHON001 | *Crotalus horridus* | Viperidae | Reptilia |
| 158 | II | CHON002 | *Crotalus horridus* | Viperidae | Reptilia |
| 159 | II | CHON004 | *Crotalus horridus* | Viperidae | Reptilia |
| 160 | II | CHON005 | *Crotalus horridus* | Viperidae | Reptilia |
| 161 | II | CHON006 | *Crotalus horridus* | Viperidae | Reptilia |
| 162 | II | CHON007 | *Crotalus horridus* | Viperidae | Reptilia |
| 163 | II | CHON008 | *Crotalus horridus* | Viperidae | Reptilia |
| 164 | II | CRAF001 | *Coelognathus radiatus* | Colubridae | Reptilia |
| 165 | II | CRAF002 | *Coelognathus radiatus* | Colubridae | Reptilia |
| 166 | II | CRAF003 | *Coelognathus radiatus* | Colubridae | Reptilia |
| 167 | II | CRAM001 | *Coelognathus radiatus* | Colubridae | Reptilia |
| 168 | II | CRAM002 | *Coelognathus radiatus* | Colubridae | Reptilia |
| 169 | II | CRAM003 | *Coelognathus radiatus* | Colubridae | Reptilia |
| 170 | II | DSIF001 | *Daboia siamensis* | Viperidae | Reptilia |
| 171 | II | DSIF002 | *Daboia siamensis* | Viperidae | Reptilia |
| 172 | II | DSIF003 | *Daboia siamensis* | Viperidae | Reptilia |
| 173 | II | DSIM001 | *Daboia siamensis* | Viperidae | Reptilia |
| 174 | II | DSIM002 | *Daboia siamensis* | Viperidae | Reptilia |
| 175 | II | DSIM003 | *Daboia siamensis* | Viperidae | Reptilia |
| 176 | II | ECON001 | *Echis coloratus* | Viperidae | Reptilia |
| 177 | II | EENF001 | *Enhydris enhydris* | Homalopsidae | Reptilia |
| 178 | II | EENF002 | *Enhydris enhydris* | Homalopsidae | Reptilia |
| 179 | II | EENF003 | *Enhydris enhydris* | Homalopsidae | Reptilia |
| 180 | II | EENM001 | *Enhydris enhydris* | Homalopsidae | Reptilia |
| 181 | II | EENM002 | *Enhydris enhydris* | Homalopsidae | Reptilia |
| 182 | II | EENM003 | *Enhydris enhydris* | Homalopsidae | Reptilia |
| 183 | II | GOXF001 | *Gonyosoma oxycephalum* | Colubridae | Reptilia |
| 184 | II | GOXF002 | *Gonyosoma oxycephalum* | Colubridae | Reptilia |
| 185 | II | GOXF003 | *Gonyosoma oxycephalum* | Colubridae | Reptilia |
| 186 | II | GOXM001 | *Gonyosoma oxycephalum* | Colubridae | Reptilia |
| 187 | II | GOXM002 | *Gonyosoma oxycephalum* | Colubridae | Reptilia |
| 188 | II | GOXM003 | *Gonyosoma oxycephalum* | Colubridae | Reptilia |
| 189 | II | HBUF001 | *Homalopsis buccata* | Homalopsidae | Reptilia |
| 190 | II | HBUF002 | *Homalopsis buccata* | Homalopsidae | Reptilia |
| 191 | II | HBUM001 | *Homalopsis buccata* | Homalopsidae | Reptilia |
| 192 | II | HBUM002 | *Homalopsis buccata* | Homalopsidae | Reptilia |
| 193 | II | NKAN001 | *Naja kaouthia* | Elapidae | Reptilia |
| 194 | II | NKAN003 | *Naja kaouthia* | Elapidae | Reptilia |
| 195 | II | NKAN004 | *Naja kaouthia* | Elapidae | Reptilia |
| 196 | II | NKAN005 | *Naja kaouthia* | Elapidae | Reptilia |
| 197 | II | NKAN006 | *Naja kaouthia* | Elapidae | Reptilia |
| 198 | II | NKAF001 | *Naja kaouthia* | Elapidae | Reptilia |
| 199 | II | NKAF002 | *Naja kaouthia* | Elapidae | Reptilia |
| 200 | II | NKAF003 | *Naja kaouthia* | Elapidae | Reptilia |
| 201 | II | NKAM002 | *Naja kaouthia* | Elapidae | Reptilia |
| 202 | II | NKAM003 | *Naja kaouthia* | Elapidae | Reptilia |
| 203 | II | NSIF001 | *Naja siamensis* | Elapidae | Reptilia |
| 204 | II | NSIF002 | *Naja siamensis* | Elapidae | Reptilia |
| 205 | II | NSIF003 | *Naja siamensis* | Elapidae | Reptilia |
| 206 | II | NSIF004 | *Naja siamensis* | Elapidae | Reptilia |
| 207 | II | NSIM001 | *Naja siamensis* | Elapidae | Reptilia |
| 208 | II | NSIM002 | *Naja siamensis* | Elapidae | Reptilia |
| 209 | II | NSIM003 | *Naja siamensis* | Elapidae | Reptilia |
| 210 | II | NSIM004 | *Naja siamensis* | Elapidae | Reptilia |
| 211 | II | NSIM005 | *Naja siamensis* | Elapidae | Reptilia |
| 212 | II | OFAF001 | *Oligodon fasciolatus* | Colubridae | Reptilia |
| 213 | II | OFAF002 | *Oligodon fasciolatus* | Colubridae | Reptilia |
| 214 | II | OFAF003 | *Oligodon fasciolatus* | Colubridae | Reptilia |
| 215 | II | OFAM001 | *Oligodon fasciolatus* | Colubridae | Reptilia |
| 216 | II | OFAM002 | *Oligodon fasciolatus* | Colubridae | Reptilia |
| 217 | II | OFAM003 | *Oligodon fasciolatus* | Colubridae | Reptilia |
| 218 | II | OHAN001 | *Ophiophagus hannah* | Elapidae | Reptilia |
| 219 | II | OHAN002 | *Ophiophagus hannah* | Elapidae | Reptilia |
| 220 | II | OHAN003 | *Ophiophagus hannah* | Elapidae | Reptilia |
| 221 | II | OHAN004 | *Ophiophagus hannah* | Elapidae | Reptilia |
| 222 | II | OHAN005 | *Ophiophagus hannah* | Elapidae | Reptilia |
| 223 | II | OHAN006 | *Ophiophagus hannah* | Elapidae | Reptilia |
| 224 | II | OHAF001 | *Ophiophagus hannah* | Elapidae | Reptilia |
| 225 | II | OHAF002 | *Ophiophagus hannah* | Elapidae | Reptilia |
| 226 | II | OHAM001 | *Ophiophagus hannah* | Elapidae | Reptilia |
| 227 | II | OHAM002 | *Ophiophagus hannah* | Elapidae | Reptilia |
| 228 | II | PFLN001 | *Protobothrops flavoviridis* | Viperidae | Reptilia |
| 229 | II | PFLN002 | *Protobothrops flavoviridis* | Viperidae | Reptilia |
| 230 | II | PFLN003 | *Protobothrops flavoviridis* | Viperidae | Reptilia |
| 231 | II | PFLN004 | *Protobothrops flavoviridis* | Viperidae | Reptilia |
| 232 | II | PFLN005 | *Protobothrops flavoviridis* | Viperidae | Reptilia |
| 233 | II | PFLN006 | *Protobothrops flavoviridis* | Viperidae | Reptilia |
| 234 | II | PFLN007 | *Protobothrops flavoviridis* | Viperidae | Reptilia |
| 235 | II | PFLN008 | *Protobothrops flavoviridis* | Viperidae | Reptilia |
| 236 | II | PFLN009 | *Protobothrops flavoviridis* | Viperidae | Reptilia |
| 237 | II | PFLN010 | *Protobothrops flavoviridis* | Viperidae | Reptilia |
| 238 | II | PGUF001 | *Pantherophis guttatus* | Colubridae | Reptilia |
| 239 | II | PGUF002 | *Pantherophis guttatus* | Colubridae | Reptilia |
| 240 | II | PGUF003 | *Pantherophis guttatus* | Colubridae | Reptilia |
| 241 | II | PGUF004 | *Pantherophis guttatus* | Colubridae | Reptilia |
| 242 | II | PGUN001 | *Pantherophis guttatus* | Colubridae | Reptilia |
| 243 | II | PGUN002 | *Pantherophis guttatus* | Colubridae | Reptilia |
| 244 | II | PGUN003 | *Pantherophis guttatus* | Colubridae | Reptilia |
| 245 | II | PGUN004 | *Pantherophis guttatus* | Colubridae | Reptilia |
| 246 | II | PGUN005 | *Pantherophis guttatus* | Colubridae | Reptilia |
| 247 | II | PGUN006 | *Pantherophis guttatus* | Colubridae | Reptilia |
| 248 | II | PGUM001 | *Pantherophis guttatus* | Colubridae | Reptilia |
| 249 | II | PGUM002 | *Pantherophis guttatus* | Colubridae | Reptilia |
| 250 | II | PGUM003 | *Pantherophis guttatus* | Colubridae | Reptilia |
| 251 | II | PGUM004 | *Pantherophis guttatus* | Colubridae | Reptilia |
| 252 | II | PMUF001 | *Ptyas mucosa* | Colubridae | Reptilia |
| 253 | II | PMUF002 | *Ptyas mucosa* | Colubridae | Reptilia |
| 254 | II | PMUM001 | *Ptyas mucosa* | Colubridae | Reptilia |
| 255 | II | PMUM002 | *Ptyas mucosa* | Colubridae | Reptilia |
| 256 | II | TSIN001 | *Thamnophis sirtalis* | Colubridae | Reptilia |
| 257 | II | TSIN002 | *Thamnophis sirtalis* | Colubridae | Reptilia |
| 258 | II | TSIN003 | *Thamnophis sirtalis* | Colubridae | Reptilia |
| 259 | II | TSIN004 | *Thamnophis sirtalis* | Colubridae | Reptilia |
| 260 | II | TSIN005 | *Thamnophis sirtalis* | Colubridae | Reptilia |
| 261 | II | TSIN006 | *Thamnophis sirtalis* | Colubridae | Reptilia |
| 262 | II | VAMN001 | *Vipera ammodytes* | Viperidae | Reptilia |
| 263 | II | VBRN001 | *Vipera berus berus* | Viperidae | Reptilia |
| 264 | II | VBRN002 | *Vipera berus berus* | Viperidae | Reptilia |
| 265 | II | VBRN003 | *Vipera berus berus* | Viperidae | Reptilia |
| 266 | II | VBRN004 | *Vipera berus berus* | Viperidae | Reptilia |
| 267 | II | VBRN005 | *Vipera berus berus* | Viperidae | Reptilia |
| 268 | II | VBRN006 | *Vipera berus berus* | Viperidae | Reptilia |
| 269 | II | VBRN007 | *Vipera berus berus* | Viperidae | Reptilia |
| 270 | II | VBRN008 | *Vipera berus berus* | Viperidae | Reptilia |
| 271 | II | XFLF001 | *Xenochrophis flavipunctatus* | Colubridae | Reptilia |
| 272 | II | XFLF002 | *Xenochrophis flavipunctatus* | Colubridae | Reptilia |
| 273 | II | XFLF003 | *Xenochrophis flavipunctatus* | Colubridae | Reptilia |
| 274 | II | XFLM001 | *Xenochrophis flavipunctatus* | Colubridae | Reptilia |
| 275 | II | XFLM002 | *Xenochrophis flavipunctatus* | Colubridae | Reptilia |
| 276 | II | XFLM003 | *Xenochrophis flavipunctatus* | Colubridae | Reptilia |
| 277 | II | EMAM004 | *Epicrates maurus* | Boidae | Reptilia |
| 278 | II | CLEN001 | *Cimex lectularius* | Cimicidae | Insecta |
| 279 | II | VSAF001 | *Varanus salvator* | Varanidae | Reptilia |
| 280 | II | VSAF002 | *Varanus salvator* | Varanidae | Reptilia |
| 281 | II | VSAF003 | *Varanus salvator* | Varanidae | Reptilia |
| 282 | II | VSAM002 | *Varanus salvator* | Varanidae | Reptilia |
