# Supplementary material for: Existence of Bov-B LINE Retrotransposons in Snake Lineages Reveals Recent Multiple Horizontal Gene Transfers with Copy Number Variation
Source: Genes (Basel). 2020 Oct 22;11(11):1241. doi: 10.3390/genes11111241 (PMC7716205; doi:10.3390/genes11111241)
Supplement: Supplementary file 1 [file genes-11-01241-s001.zip › supplementary table/Table S4.docx]

**Table S4.** Pairwise comparison of nucleotide sequence divergences (*p*-distance) of BovB retroelement among thirty-two snake species.

| *p*-distance | Abbrevation | ACO | | | AJA | | | APR | | | BCA | | | BCO | | | BDE | | | BFL | | |
| --- | --- | --- | --- | --- | --- | --- | --- | --- | --- | --- | --- | --- | --- | --- | --- | --- | --- | --- | --- | --- | --- | --- |
| *Agkistrodon contortrix* | ACO |  |  |  |  |  |  |  |  |  |  |  |  |  |  |  |  |  |  |  |  |  |
| *Acrochordus javanicus* | AJA | 0.24 | ± | 0.06 |  |  |  |  |  |  |  |  |  |  |  |  |  |  |  |  |  |  |
| *Ahaetulla prasina* | APR | 0.18 | ± | 0.05 | 0.10 | ± | 0.03 |  |  |  |  |  |  |  |  |  |  |  |  |  |  |  |
| *Bungarus candidus* | BCA | 0.20 | ± | 0.05 | 0.12 | ± | 0.03 | 0.06 | ± | 0.02 |  |  |  |  |  |  |  |  |  |  |  |  |
| *Boa constrictor* | BCO | 0.18 | ± | 0.05 | 0.08 | ± | 0.03 | 0.04 | ± | 0.02 | 0.06 | ± | 0.02 |  |  |  |  |  |  |  |  |  |
| *Boiga dendrophila* | BDE | 0.20 | ± | 0.05 | 0.13 | ± | 0.03 | 0.08 | ± | 0.01 | 0.10 | ± | 0.02 | 0.07 | ± | 0.02 |  |  |  |  |  |  |
| *Bungarus flaviceps* | BFL | 0.22 | ± | 0.05 | 0.14 | ± | 0.03 | 0.09 | ± | 0.02 | 0.11 | ± | 0.02 | 0.08 | ± | 0.02 | 0.12 | ± | 0.02 |  |  |  |
| *Coelognathus flavolineatus* | CFL | 0.21 | ± | 0.05 | 0.13 | ± | 0.03 | 0.07 | ± | 0.01 | 0.09 | ± | 0.02 | 0.07 | ± | 0.02 | 0.10 | ± | 0.02 | 0.11 | ± | 0.02 |
| *Crotalus horridus* | CHO | 0.21 | ± | 0.05 | 0.12 | ± | 0.03 | 0.07 | ± | 0.01 | 0.09 | ± | 0.02 | 0.06 | ± | 0.02 | 0.10 | ± | 0.01 | 0.11 | ± | 0.02 |
| *Crotalus pyrrhus* | CPY | 0.18 | ± | 0.05 | 0.11 | ± | 0.04 | 0.06 | ± | 0.03 | 0.08 | ± | 0.03 | 0.05 | ± | 0.03 | 0.09 | ± | 0.03 | 0.10 | ± | 0.03 |
| *Coelognathus radiatus* | CRA | 0.20 | ± | 0.05 | 0.12 | ± | 0.03 | 0.06 | ± | 0.02 | 0.08 | ± | 0.02 | 0.06 | ± | 0.02 | 0.10 | ± | 0.02 | 0.11 | ± | 0.02 |
| *Cylindrophis ruffus* | CRU | 0.22 | ± | 0.05 | 0.11 | ± | 0.03 | 0.08 | ± | 0.03 | 0.10 | ± | 0.03 | 0.05 | ± | 0.02 | 0.11 | ± | 0.02 | 0.12 | ± | 0.03 |
| *Daboia siamensis* | DSI | 0.20 | ± | 0.05 | 0.12 | ± | 0.03 | 0.07 | ± | 0.02 | 0.09 | ± | 0.02 | 0.06 | ± | 0.02 | 0.11 | ± | 0.02 | 0.12 | ± | 0.02 |
| *Echis coloratus* | ECO | 0.18 | ± | 0.05 | 0.10 | ± | 0.04 | 0.04 | ± | 0.02 | 0.06 | ± | 0.02 | 0.04 | ± | 0.03 | 0.08 | ± | 0.02 | 0.09 | ± | 0.02 |
| *Enhydris enhydris* | EEN | 0.22 | ± | 0.05 | 0.12 | ± | 0.03 | 0.07 | ± | 0.02 | 0.09 | ± | 0.02 | 0.06 | ± | 0.02 | 0.11 | ± | 0.02 | 0.12 | ± | 0.02 |
| *Epicrates maurus* | EMA | 0.21 | ± | 0.05 | 0.11 | ± | 0.03 | 0.07 | ± | 0.02 | 0.09 | ± | 0.02 | 0.04 | ± | 0.01 | 0.10 | ± | 0.02 | 0.11 | ± | 0.02 |
| *Gonyosoma oxycephalum* | GOX | 0.19 | ± | 0.05 | 0.10 | ± | 0.03 | 0.05 | ± | 0.01 | 0.07 | ± | 0.02 | 0.05 | ± | 0.02 | 0.09 | ± | 0.01 | 0.10 | ± | 0.02 |
| *Homalopsis buccata* | HBU | 0.20 | ± | 0.05 | 0.11 | ± | 0.03 | 0.06 | ± | 0.01 | 0.08 | ± | 0.02 | 0.04 | ± | 0.01 | 0.09 | ± | 0.01 | 0.10 | ± | 0.02 |
| *Naja kaouthia* | NKA | 0.18 | ± | 0.05 | 0.11 | ± | 0.03 | 0.06 | ± | 0.01 | 0.08 | ± | 0.01 | 0.05 | ± | 0.02 | 0.09 | ± | 0.01 | 0.10 | ± | 0.02 |
| *Naja siamensis* | NSI | 0.20 | ± | 0.05 | 0.12 | ± | 0.03 | 0.07 | ± | 0.01 | 0.09 | ± | 0.02 | 0.07 | ± | 0.02 | 0.10 | ± | 0.02 | 0.11 | ± | 0.02 |
| *Oligodon fasciolatus* | OFA | 0.20 | ± | 0.05 | 0.11 | ± | 0.03 | 0.06 | ± | 0.01 | 0.08 | ± | 0.01 | 0.05 | ± | 0.02 | 0.09 | ± | 0.01 | 0.10 | ± | 0.02 |
| *Ophiophagus hannah* | OHA | 0.18 | ± | 0.05 | 0.10 | ± | 0.03 | 0.05 | ± | 0.01 | 0.07 | ± | 0.01 | 0.05 | ± | 0.02 | 0.09 | ± | 0.01 | 0.09 | ± | 0.02 |
| *Python bivittatus* | PBI | 0.21 | ± | 0.05 | 0.10 | ± | 0.03 | 0.06 | ± | 0.02 | 0.09 | ± | 0.02 | 0.03 | ± | 0.01 | 0.09 | ± | 0.02 | 0.10 | ± | 0.02 |
| *Protobothrops flavoviridis* | PFL | 0.20 | ± | 0.05 | 0.11 | ± | 0.03 | 0.05 | ± | 0.02 | 0.07 | ± | 0.02 | 0.05 | ± | 0.02 | 0.09 | ± | 0.02 | 0.10 | ± | 0.02 |
| *Pantherophis guttatus* | PGU | 0.19 | ± | 0.05 | 0.11 | ± | 0.03 | 0.05 | ± | 0.01 | 0.07 | ± | 0.01 | 0.05 | ± | 0.02 | 0.09 | ± | 0.01 | 0.10 | ± | 0.01 |
| *Ptyas mucosa* | PMU | 0.20 | ± | 0.05 | 0.12 | ± | 0.03 | 0.06 | ± | 0.02 | 0.08 | ± | 0.02 | 0.05 | ± | 0.02 | 0.10 | ± | 0.02 | 0.11 | ± | 0.02 |
| *Python regius* | PRE | 0.21 | ± | 0.05 | 0.10 | ± | 0.03 | 0.07 | ± | 0.02 | 0.09 | ± | 0.03 | 0.03 | ± | 0.02 | 0.10 | ± | 0.02 | 0.10 | ± | 0.03 |
| *Thamnophis sirtalis* | TSI | 0.19 | ± | 0.05 | 0.11 | ± | 0.03 | 0.06 | ± | 0.01 | 0.08 | ± | 0.02 | 0.05 | ± | 0.02 | 0.09 | ± | 0.01 | 0.10 | ± | 0.02 |
| *Vipera ammodytes* | VAM | 0.18 | ± | 0.05 | 0.10 | ± | 0.04 | 0.04 | ± | 0.02 | 0.06 | ± | 0.02 | 0.04 | ± | 0.03 | 0.08 | ± | 0.02 | 0.09 | ± | 0.02 |
| *Vipera berus berus* | VBR | 0.19 | ± | 0.05 | 0.10 | ± | 0.03 | 0.05 | ± | 0.01 | 0.07 | ± | 0.02 | 0.04 | ± | 0.02 | 0.09 | ± | 0.01 | 0.10 | ± | 0.02 |
| *Xenochrophis flavipunctatus* | XFL | 0.21 | ± | 0.05 | 0.14 | ± | 0.03 | 0.09 | ± | 0.02 | 0.10 | ± | 0.02 | 0.08 | ± | 0.02 | 0.12 | ± | 0.02 | 0.12 | ± | 0.02 |
| *Xenopeltis unicolor* | XUN | 0.22 | ± | 0.05 | 0.12 | ± | 0.03 | 0.10 | ± | 0.02 | 0.11 | ± | 0.03 | 0.06 | ± | 0.02 | 0.12 | ± | 0.02 | 0.13 | ± | 0.03 |

**Table S2 (continuous)**

| *p*-distance | Abbrevation | CHO | | | CPY | | | CRA | | | CRU | | | DSI | | | ECO | | | EEN | | |
| --- | --- | --- | --- | --- | --- | --- | --- | --- | --- | --- | --- | --- | --- | --- | --- | --- | --- | --- | --- | --- | --- | --- |
| *Agkistrodon contortrix* | ACO |  |  |  |  |  |  |  |  |  |  |  |  |  |  |  |  |  |  |  |  |  |
| *Acrochordus javanicus* | AJA |  |  |  |  |  |  |  |  |  |  |  |  |  |  |  |  |  |  |  |  |  |
| *Ahaetulla prasina* | APR |  |  |  |  |  |  |  |  |  |  |  |  |  |  |  |  |  |  |  |  |  |
| *Bungarus candidus* | BCA |  |  |  |  |  |  |  |  |  |  |  |  |  |  |  |  |  |  |  |  |  |
| *Boa constrictor* | BCO |  |  |  |  |  |  |  |  |  |  |  |  |  |  |  |  |  |  |  |  |  |
| *Boiga dendrophila* | BDE |  |  |  |  |  |  |  |  |  |  |  |  |  |  |  |  |  |  |  |  |  |
| *Bungarus flaviceps* | BFL |  |  |  |  |  |  |  |  |  |  |  |  |  |  |  |  |  |  |  |  |  |
| *Coelognathus flavolineatus* | CFL |  |  |  |  |  |  |  |  |  |  |  |  |  |  |  |  |  |  |  |  |  |
| *Crotalus horridus* | CHO |  |  |  |  |  |  |  |  |  |  |  |  |  |  |  |  |  |  |  |  |  |
| *Crotalus pyrrhus* | CPY | 0.08 | ± | 0.03 |  |  |  |  |  |  |  |  |  |  |  |  |  |  |  |  |  |  |
| *Coelognathus radiatus* | CRA | 0.09 | ± | 0.02 | 0.08 | ± | 0.03 |  |  |  |  |  |  |  |  |  |  |  |  |  |  |  |
| *Cylindrophis ruffus* | CRU | 0.10 | ± | 0.03 | 0.10 | ± | 0.03 | 0.10 | ± | 0.03 |  |  |  |  |  |  |  |  |  |  |  |  |
| *Daboia siamensis* | DSI | 0.10 | ± | 0.02 | 0.09 | ± | 0.03 | 0.09 | ± | 0.02 | 0.10 | ± | 0.03 |  |  |  |  |  |  |  |  |  |
| *Echis coloratus* | ECO | 0.07 | ± | 0.02 | 0.05 | ± | 0.03 | 0.06 | ± | 0.02 | 0.08 | ± | 0.03 | 0.04 | ± | 0.01 |  |  |  |  |  |  |
| *Enhydris enhydris* | EEN | 0.10 | ± | 0.02 | 0.09 | ± | 0.03 | 0.09 | ± | 0.02 | 0.10 | ± | 0.02 | 0.10 | ± | 0.02 | 0.07 | ± | 0.02 |  |  |  |
| *Epicrates maurus* | EMA | 0.09 | ± | 0.02 | 0.08 | ± | 0.03 | 0.10 | ± | 0.02 | 0.08 | ± | 0.02 | 0.09 | ± | 0.03 | 0.07 | ± | 0.03 | 0.10 | ± | 0.02 |
| *Gonyosoma oxycephalum* | GOX | 0.07 | ± | 0.01 | 0.07 | ± | 0.03 | 0.07 | ± | 0.02 | 0.08 | ± | 0.03 | 0.08 | ± | 0.02 | 0.05 | ± | 0.02 | 0.08 | ± | 0.02 |
| *Homalopsis buccata* | HBU | 0.08 | ± | 0.02 | 0.07 | ± | 0.03 | 0.08 | ± | 0.02 | 0.09 | ± | 0.02 | 0.09 | ± | 0.02 | 0.06 | ± | 0.02 | 0.09 | ± | 0.02 |
| *Naja kaouthia* | NKA | 0.08 | ± | 0.01 | 0.06 | ± | 0.02 | 0.07 | ± | 0.02 | 0.09 | ± | 0.02 | 0.08 | ± | 0.02 | 0.05 | ± | 0.02 | 0.08 | ± | 0.01 |
| *Naja siamensis* | NSI | 0.09 | ± | 0.02 | 0.08 | ± | 0.03 | 0.09 | ± | 0.02 | 0.11 | ± | 0.02 | 0.10 | ± | 0.02 | 0.07 | ± | 0.02 | 0.10 | ± | 0.02 |
| *Oligodon fasciolatus* | OFA | 0.08 | ± | 0.01 | 0.07 | ± | 0.02 | 0.08 | ± | 0.02 | 0.09 | ± | 0.02 | 0.09 | ± | 0.02 | 0.06 | ± | 0.02 | 0.09 | ± | 0.01 |
| *Ophiophagus hannah* | OHA | 0.08 | ± | 0.01 | 0.06 | ± | 0.02 | 0.07 | ± | 0.02 | 0.08 | ± | 0.02 | 0.08 | ± | 0.02 | 0.05 | ± | 0.02 | 0.08 | ± | 0.01 |
| *Python bivittatus* | PBI | 0.08 | ± | 0.02 | 0.08 | ± | 0.03 | 0.08 | ± | 0.02 | 0.07 | ± | 0.02 | 0.09 | ± | 0.03 | 0.06 | ± | 0.03 | 0.09 | ± | 0.02 |
| *Protobothrops flavoviridis* | PFL | 0.07 | ± | 0.02 | 0.07 | ± | 0.03 | 0.07 | ± | 0.02 | 0.09 | ± | 0.03 | 0.08 | ± | 0.02 | 0.05 | ± | 0.02 | 0.09 | ± | 0.02 |
| *Pantherophis guttatus* | PGU | 0.08 | ± | 0.01 | 0.07 | ± | 0.02 | 0.08 | ± | 0.01 | 0.09 | ± | 0.02 | 0.08 | ± | 0.02 | 0.05 | ± | 0.02 | 0.09 | ± | 0.01 |
| *Ptyas mucosa* | PMU | 0.09 | ± | 0.02 | 0.08 | ± | 0.03 | 0.08 | ± | 0.02 | 0.10 | ± | 0.03 | 0.10 | ± | 0.02 | 0.06 | ± | 0.02 | 0.09 | ± | 0.02 |
| *Python regius* | PRE | 0.09 | ± | 0.02 | 0.08 | ± | 0.03 | 0.09 | ± | 0.03 | 0.07 | ± | 0.02 | 0.09 | ± | 0.03 | 0.06 | ± | 0.03 | 0.09 | ± | 0.02 |
| *Thamnophis sirtalis* | TSI | 0.08 | ± | 0.01 | 0.08 | ± | 0.03 | 0.08 | ± | 0.02 | 0.09 | ± | 0.03 | 0.09 | ± | 0.02 | 0.05 | ± | 0.02 | 0.09 | ± | 0.02 |
| *Vipera ammodytes* | VAM | 0.07 | ± | 0.02 | 0.05 | ± | 0.03 | 0.06 | ± | 0.02 | 0.08 | ± | 0.03 | 0.04 | ± | 0.01 | 0.00 | ± | 0.00 | 0.07 | ± | 0.02 |
| *Vipera berus berus* | VBR | 0.07 | ± | 0.01 | 0.06 | ± | 0.02 | 0.07 | ± | 0.01 | 0.08 | ± | 0.02 | 0.08 | ± | 0.02 | 0.04 | ± | 0.02 | 0.08 | ± | 0.01 |
| *Xenochrophis flavipunctatus* | XFL | 0.11 | ± | 0.02 | 0.09 | ± | 0.03 | 0.10 | ± | 0.02 | 0.12 | ± | 0.03 | 0.12 | ± | 0.02 | 0.08 | ± | 0.02 | 0.11 | ± | 0.02 |
| *Xenopeltis unicolor* | XUN | 0.11 | ± | 0.02 | 0.10 | ± | 0.03 | 0.11 | ± | 0.03 | 0.10 | ± | 0.02 | 0.12 | ± | 0.03 | 0.09 | ± | 0.03 | 0.12 | ± | 0.02 |

**Table S2 (continuous)**

| *p*-distance | Abbrevation | EMA | | | GOX | | | HBU | | | NKA | | | NSI | | | OFA | | | OHA | | |
| --- | --- | --- | --- | --- | --- | --- | --- | --- | --- | --- | --- | --- | --- | --- | --- | --- | --- | --- | --- | --- | --- | --- |
| *Agkistrodon contortrix* | ACO |  |  |  |  |  |  |  |  |  |  |  |  |  |  |  |  |  |  |  |  |  |
| *Acrochordus javanicus* | AJA |  |  |  |  |  |  |  |  |  |  |  |  |  |  |  |  |  |  |  |  |  |
| *Ahaetulla prasina* | APR |  |  |  |  |  |  |  |  |  |  |  |  |  |  |  |  |  |  |  |  |  |
| *Bungarus candidus* | BCA |  |  |  |  |  |  |  |  |  |  |  |  |  |  |  |  |  |  |  |  |  |
| *Boa constrictor* | BCO |  |  |  |  |  |  |  |  |  |  |  |  |  |  |  |  |  |  |  |  |  |
| *Boiga dendrophila* | BDE |  |  |  |  |  |  |  |  |  |  |  |  |  |  |  |  |  |  |  |  |  |
| *Bungarus flaviceps* | BFL |  |  |  |  |  |  |  |  |  |  |  |  |  |  |  |  |  |  |  |  |  |
| *Coelognathus flavolineatus* | CFL |  |  |  |  |  |  |  |  |  |  |  |  |  |  |  |  |  |  |  |  |  |
| *Crotalus horridus* | CHO |  |  |  |  |  |  |  |  |  |  |  |  |  |  |  |  |  |  |  |  |  |
| *Crotalus pyrrhus* | CPY |  |  |  |  |  |  |  |  |  |  |  |  |  |  |  |  |  |  |  |  |  |
| *Coelognathus radiatus* | CRA |  |  |  |  |  |  |  |  |  |  |  |  |  |  |  |  |  |  |  |  |  |
| *Cylindrophis ruffus* | CRU |  |  |  |  |  |  |  |  |  |  |  |  |  |  |  |  |  |  |  |  |  |
| *Daboia siamensis* | DSI |  |  |  |  |  |  |  |  |  |  |  |  |  |  |  |  |  |  |  |  |  |
| *Echis coloratus* | ECO |  |  |  |  |  |  |  |  |  |  |  |  |  |  |  |  |  |  |  |  |  |
| *Enhydris enhydris* | EEN |  |  |  |  |  |  |  |  |  |  |  |  |  |  |  |  |  |  |  |  |  |
| *Epicrates maurus* | EMA |  |  |  |  |  |  |  |  |  |  |  |  |  |  |  |  |  |  |  |  |  |
| *Gonyosoma oxycephalum* | GOX | 0.08 | ± | 0.02 |  |  |  |  |  |  |  |  |  |  |  |  |  |  |  |  |  |  |
| *Homalopsis buccata* | HBU | 0.08 | ± | 0.02 | 0.07 | ± | 0.01 |  |  |  |  |  |  |  |  |  |  |  |  |  |  |  |
| *Naja kaouthia* | NKA | 0.08 | ± | 0.02 | 0.06 | ± | 0.01 | 0.07 | ± | 0.01 |  |  |  |  |  |  |  |  |  |  |  |  |
| *Naja siamensis* | NSI | 0.10 | ± | 0.02 | 0.08 | ± | 0.01 | 0.09 | ± | 0.02 | 0.08 | ± | 0.02 |  |  |  |  |  |  |  |  |  |
| *Oligodon fasciolatus* | OFA | 0.09 | ± | 0.02 | 0.07 | ± | 0.01 | 0.07 | ± | 0.01 | 0.07 | ± | 0.01 | 0.09 | ± | 0.02 |  |  |  |  |  |  |
| *Ophiophagus hannah* | OHA | 0.08 | ± | 0.02 | 0.06 | ± | 0.01 | 0.07 | ± | 0.01 | 0.06 | ± | 0.01 | 0.08 | ± | 0.01 | 0.07 | ± | 0.01 |  |  |  |
| *Python bivittatus* | PBI | 0.06 | ± | 0.01 | 0.07 | ± | 0.02 | 0.07 | ± | 0.02 | 0.07 | ± | 0.02 | 0.09 | ± | 0.02 | 0.07 | ± | 0.02 | 0.07 | ± | 0.02 |
| *Protobothrops flavoviridis* | PFL | 0.08 | ± | 0.02 | 0.06 | ± | 0.02 | 0.07 | ± | 0.02 | 0.07 | ± | 0.02 | 0.08 | ± | 0.02 | 0.07 | ± | 0.02 | 0.06 | ± | 0.02 |
| *Pantherophis guttatus* | PGU | 0.09 | ± | 0.02 | 0.06 | ± | 0.01 | 0.07 | ± | 0.01 | 0.07 | ± | 0.01 | 0.08 | ± | 0.01 | 0.07 | ± | 0.01 | 0.06 | ± | 0.01 |
| *Ptyas mucosa* | PMU | 0.09 | ± | 0.02 | 0.07 | ± | 0.02 | 0.08 | ± | 0.02 | 0.08 | ± | 0.02 | 0.09 | ± | 0.02 | 0.08 | ± | 0.02 | 0.07 | ± | 0.02 |
| *Python regius* | PRE | 0.06 | ± | 0.02 | 0.07 | ± | 0.03 | 0.07 | ± | 0.02 | 0.07 | ± | 0.02 | 0.09 | ± | 0.02 | 0.08 | ± | 0.02 | 0.07 | ± | 0.02 |
| *Thamnophis sirtalis* | TSI | 0.09 | ± | 0.02 | 0.07 | ± | 0.01 | 0.08 | ± | 0.02 | 0.07 | ± | 0.01 | 0.09 | ± | 0.02 | 0.08 | ± | 0.01 | 0.07 | ± | 0.01 |
| *Vipera ammodytes* | VAM | 0.07 | ± | 0.03 | 0.05 | ± | 0.02 | 0.06 | ± | 0.02 | 0.05 | ± | 0.02 | 0.07 | ± | 0.02 | 0.06 | ± | 0.02 | 0.05 | ± | 0.02 |
| *Vipera berus berus* | VBR | 0.08 | ± | 0.02 | 0.06 | ± | 0.01 | 0.07 | ± | 0.01 | 0.06 | ± | 0.01 | 0.08 | ± | 0.01 | 0.06 | ± | 0.01 | 0.06 | ± | 0.01 |
| *Xenochrophis flavipunctatus* | XFL | 0.12 | ± | 0.02 | 0.09 | ± | 0.02 | 0.10 | ± | 0.02 | 0.10 | ± | 0.02 | 0.11 | ± | 0.02 | 0.10 | ± | 0.02 | 0.10 | ± | 0.02 |
| *Xenopeltis unicolor* | XUN | 0.09 | ± | 0.02 | 0.10 | ± | 0.02 | 0.10 | ± | 0.02 | 0.10 | ± | 0.02 | 0.12 | ± | 0.02 | 0.10 | ± | 0.02 | 0.10 | ± | 0.02 |

**Table S2 (continuous)**

| *p*-distance | Abbrevation | PBI | | | PFL | | | PGU | | | PMU | | | PRE | | | TSI | | | VAM | | |
| --- | --- | --- | --- | --- | --- | --- | --- | --- | --- | --- | --- | --- | --- | --- | --- | --- | --- | --- | --- | --- | --- | --- |
| *Agkistrodon contortrix* | ACO |  |  |  |  |  |  |  |  |  |  |  |  |  |  |  |  |  |  |  |  |  |
| *Acrochordus javanicus* | AJA |  |  |  |  |  |  |  |  |  |  |  |  |  |  |  |  |  |  |  |  |  |
| *Ahaetulla prasina* | APR |  |  |  |  |  |  |  |  |  |  |  |  |  |  |  |  |  |  |  |  |  |
| *Bungarus candidus* | BCA |  |  |  |  |  |  |  |  |  |  |  |  |  |  |  |  |  |  |  |  |  |
| *Boa constrictor* | BCO |  |  |  |  |  |  |  |  |  |  |  |  |  |  |  |  |  |  |  |  |  |
| *Boiga dendrophila* | BDE |  |  |  |  |  |  |  |  |  |  |  |  |  |  |  |  |  |  |  |  |  |
| *Bungarus flaviceps* | BFL |  |  |  |  |  |  |  |  |  |  |  |  |  |  |  |  |  |  |  |  |  |
| *Coelognathus flavolineatus* | CFL |  |  |  |  |  |  |  |  |  |  |  |  |  |  |  |  |  |  |  |  |  |
| *Crotalus horridus* | CHO |  |  |  |  |  |  |  |  |  |  |  |  |  |  |  |  |  |  |  |  |  |
| *Crotalus pyrrhus* | CPY |  |  |  |  |  |  |  |  |  |  |  |  |  |  |  |  |  |  |  |  |  |
| *Coelognathus radiatus* | CRA |  |  |  |  |  |  |  |  |  |  |  |  |  |  |  |  |  |  |  |  |  |
| *Cylindrophis ruffus* | CRU |  |  |  |  |  |  |  |  |  |  |  |  |  |  |  |  |  |  |  |  |  |
| *Daboia siamensis* | DSI |  |  |  |  |  |  |  |  |  |  |  |  |  |  |  |  |  |  |  |  |  |
| *Echis coloratus* | ECO |  |  |  |  |  |  |  |  |  |  |  |  |  |  |  |  |  |  |  |  |  |
| *Enhydris enhydris* | EEN |  |  |  |  |  |  |  |  |  |  |  |  |  |  |  |  |  |  |  |  |  |
| *Epicrates maurus* | EMA |  |  |  |  |  |  |  |  |  |  |  |  |  |  |  |  |  |  |  |  |  |
| *Gonyosoma oxycephalum* | GOX |  |  |  |  |  |  |  |  |  |  |  |  |  |  |  |  |  |  |  |  |  |
| *Homalopsis buccata* | HBU |  |  |  |  |  |  |  |  |  |  |  |  |  |  |  |  |  |  |  |  |  |
| *Naja kaouthia* | NKA |  |  |  |  |  |  |  |  |  |  |  |  |  |  |  |  |  |  |  |  |  |
| *Naja siamensis* | NSI |  |  |  |  |  |  |  |  |  |  |  |  |  |  |  |  |  |  |  |  |  |
| *Oligodon fasciolatus* | OFA |  |  |  |  |  |  |  |  |  |  |  |  |  |  |  |  |  |  |  |  |  |
| *Ophiophagus hannah* | OHA |  |  |  |  |  |  |  |  |  |  |  |  |  |  |  |  |  |  |  |  |  |
| *Python bivittatus* | PBI |  |  |  |  |  |  |  |  |  |  |  |  |  |  |  |  |  |  |  |  |  |
| *Protobothrops flavoviridis* | PFL | 0.08 | ± | 0.02 |  |  |  |  |  |  |  |  |  |  |  |  |  |  |  |  |  |  |
| *Pantherophis guttatus* | PGU | 0.08 | ± | 0.02 | 0.07 | ± | 0.02 |  |  |  |  |  |  |  |  |  |  |  |  |  |  |  |
| *Ptyas mucosa* | PMU | 0.08 | ± | 0.02 | 0.08 | ± | 0.02 | 0.08 | ± | 0.01 |  |  |  |  |  |  |  |  |  |  |  |  |
| *Python regius* | PRE | 0.04 | ± | 0.01 | 0.08 | ± | 0.03 | 0.08 | ± | 0.02 | 0.08 | ± | 0.02 |  |  |  |  |  |  |  |  |  |
| *Thamnophis sirtalis* | TSI | 0.08 | ± | 0.02 | 0.07 | ± | 0.02 | 0.07 | ± | 0.01 | 0.08 | ± | 0.02 | 0.08 | ± | 0.02 |  |  |  |  |  |  |
| *Vipera ammodytes* | VAM | 0.06 | ± | 0.03 | 0.05 | ± | 0.02 | 0.05 | ± | 0.02 | 0.06 | ± | 0.02 | 0.06 | ± | 0.03 | 0.05 | ± | 0.02 |  |  |  |
| *Vipera berus berus* | VBR | 0.07 | ± | 0.02 | 0.06 | ± | 0.02 | 0.06 | ± | 0.01 | 0.07 | ± | 0.02 | 0.07 | ± | 0.02 | 0.07 | ± | 0.01 | 0.04 | ± | 0.02 |
| *Xenochrophis flavipunctatus* | XFL | 0.11 | ± | 0.02 | 0.10 | ± | 0.02 | 0.10 | ± | 0.02 | 0.10 | ± | 0.02 | 0.11 | ± | 0.03 | 0.10 | ± | 0.02 | 0.08 | ± | 0.02 |
| *Xenopeltis unicolor* | XUN | 0.08 | ± | 0.02 | 0.11 | ± | 0.03 | 0.11 | ± | 0.02 | 0.11 | ± | 0.02 | 0.08 | ± | 0.02 | 0.11 | ± | 0.02 | 0.09 | ± | 0.03 |

**Table S2 (continuous)**

| *p*-distance | Abbrevation | VBR | | | XFL | | | XUN | | |
| --- | --- | --- | --- | --- | --- | --- | --- | --- | --- | --- |
| *Agkistrodon contortrix* | ACO |  |  |  |  |  |  |  |  |  |
| *Acrochordus javanicus* | AJA |  |  |  |  |  |  |  |  |  |
| *Ahaetulla prasina* | APR |  |  |  |  |  |  |  |  |  |
| *Bungarus candidus* | BCA |  |  |  |  |  |  |  |  |  |
| *Boa constrictor* | BCO |  |  |  |  |  |  |  |  |  |
| *Boiga dendrophila* | BDE |  |  |  |  |  |  |  |  |  |
| *Bungarus flaviceps* | BFL |  |  |  |  |  |  |  |  |  |
| *Coelognathus flavolineatus* | CFL |  |  |  |  |  |  |  |  |  |
| *Crotalus horridus* | CHO |  |  |  |  |  |  |  |  |  |
| *Crotalus pyrrhus* | CPY |  |  |  |  |  |  |  |  |  |
| *Coelognathus radiatus* | CRA |  |  |  |  |  |  |  |  |  |
| *Cylindrophis ruffus* | CRU |  |  |  |  |  |  |  |  |  |
| *Daboia siamensis* | DSI |  |  |  |  |  |  |  |  |  |
| *Echis coloratus* | ECO |  |  |  |  |  |  |  |  |  |
| *Enhydris enhydris* | EEN |  |  |  |  |  |  |  |  |  |
| *Epicrates maurus* | EMA |  |  |  |  |  |  |  |  |  |
| *Gonyosoma oxycephalum* | GOX |  |  |  |  |  |  |  |  |  |
| *Homalopsis buccata* | HBU |  |  |  |  |  |  |  |  |  |
| *Naja kaouthia* | NKA |  |  |  |  |  |  |  |  |  |
| *Naja siamensis* | NSI |  |  |  |  |  |  |  |  |  |
| *Oligodon fasciolatus* | OFA |  |  |  |  |  |  |  |  |  |
| *Ophiophagus hannah* | OHA |  |  |  |  |  |  |  |  |  |
| *Python bivittatus* | PBI |  |  |  |  |  |  |  |  |  |
| *Protobothrops flavoviridis* | PFL |  |  |  |  |  |  |  |  |  |
| *Pantherophis guttatus* | PGU |  |  |  |  |  |  |  |  |  |
| *Ptyas mucosa* | PMU |  |  |  |  |  |  |  |  |  |
| *Python regius* | PRE |  |  |  |  |  |  |  |  |  |
| *Thamnophis sirtalis* | TSI |  |  |  |  |  |  |  |  |  |
| *Vipera ammodytes* | VAM |  |  |  |  |  |  |  |  |  |
| *Vipera berus berus* | VBR |  |  |  |  |  |  |  |  |  |
| *Xenochrophis flavipunctatus* | XFL | 0.09 | ± | 0.02 |  |  |  |  |  | 0.09 |
| *Xenopeltis unicolor* | XUN | 0.10 | ± | 0.02 | 0.13 | ± | 0.03 |  |  | 0.10 |
