# Supplementary material for: Existence of Bov-B LINE Retrotransposons in Snake Lineages Reveals Recent Multiple Horizontal Gene Transfers with Copy Number Variation
Source: Genes (Basel). 2020 Oct 22;11(11):1241. doi: 10.3390/genes11111241 (PMC7716205; doi:10.3390/genes11111241)
Supplement: Supplementary file 1 [file genes-11-01241-s001.zip › supplementary table/Table S5.docx]

**Table S5.** Nonsynonymous substitution sites (*K_a_*) per Synonymous substitution site (*K_s_*) of BovB retroelement among thirty-two snake species.

|  | K_a_/K_s_ | Abbrevation | ACO | AJA | APR | BCA | BCO | BDE | BFL | CFL | CHO | CPY | CRA | CRU | DSI | ECO | EEN | EMA | GOX | HBU | NKA | NSI | OFA | OHA | PBI | PFL | PGU | PMU | PRE | TSI | VAM | VBR | XFL | XUN |
| --- | --- | --- | --- | --- | --- | --- | --- | --- | --- | --- | --- | --- | --- | --- | --- | --- | --- | --- | --- | --- | --- | --- | --- | --- | --- | --- | --- | --- | --- | --- | --- | --- | --- | --- |
| 1 | *Agkistrodon contortrix* | ACO |  |  |  |  |  |  |  |  |  |  |  |  |  |  |  |  |  |  |  |  |  |  |  |  |  |  |  |  |  |  |  |  |
| 2 | *Acrochordus javanicus* | AJA | 0.69 |  |  |  |  |  |  |  |  |  |  |  |  |  |  |  |  |  |  |  |  |  |  |  |  |  |  |  |  |  |  |  |
| 3 | *Ahaetulla prasina* | APR | 1.34 | 0.43 |  |  |  |  |  |  |  |  |  |  |  |  |  |  |  |  |  |  |  |  |  |  |  |  |  |  |  |  |  |  |
| 4 | *Bungarus candidus* | BCA | 0.94 | 0.43 | 0.59 |  |  |  |  |  |  |  |  |  |  |  |  |  |  |  |  |  |  |  |  |  |  |  |  |  |  |  |  |  |
| 5 | *Boa constrictor* | BCO | 0.69 | 0.29 | 0.49 | 0.46 |  |  |  |  |  |  |  |  |  |  |  |  |  |  |  |  |  |  |  |  |  |  |  |  |  |  |  |  |
| 6 | *Boiga dendrophila* | BDE | 1.12 | 0.49 | 0.84 | 0.69 | 0.62 |  |  |  |  |  |  |  |  |  |  |  |  |  |  |  |  |  |  |  |  |  |  |  |  |  |  |  |
| 7 | *Bungarus flaviceps* | BFL | 0.95 | 0.53 | 0.75 | 0.86 | 0.67 | 0.86 |  |  |  |  |  |  |  |  |  |  |  |  |  |  |  |  |  |  |  |  |  |  |  |  |  |  |
| 8 | *Coelognathus flavolineatus* | CFL | 1.07 | 0.51 | 0.80 | 0.82 | 0.65 | 0.82 | 0.87 |  |  |  |  |  |  |  |  |  |  |  |  |  |  |  |  |  |  |  |  |  |  |  |  |  |
| 9 | *Crotalus horridus* | CHO | 1.04 | 0.38 | 0.79 | 0.84 | 0.42 | 0.84 | 0.77 | 0.76 |  |  |  |  |  |  |  |  |  |  |  |  |  |  |  |  |  |  |  |  |  |  |  |  |
| 10 | *Crotalus pyrrhus* | CPY | 1.09 | 0.50 | 0.88 | 1.04 | 0.45 | 1.04 | 1.06 | 1.04 | 0.88 |  |  |  |  |  |  |  |  |  |  |  |  |  |  |  |  |  |  |  |  |  |  |  |
| 11 | *Coelognathus radiatus* | CRA | 1.04 | 0.39 | 0.65 | 0.72 | 0.45 | 0.72 | 0.72 | 0.69 | 0.64 | 0.88 |  |  |  |  |  |  |  |  |  |  |  |  |  |  |  |  |  |  |  |  |  |  |
| 12 | *Cylindrophis ruffus* | CRU | 0.62 | 0.37 | 0.39 | 0.42 | 0.28 | 0.42 | 0.46 | 0.46 | 0.36 | 0.52 | 0.37 |  |  |  |  |  |  |  |  |  |  |  |  |  |  |  |  |  |  |  |  |  |
| 13 | *Daboia siamensis* | DSI | 1.01 | 0.45 | 0.79 | 0.77 | 0.63 | 0.77 | 0.70 | 0.76 | 0.63 | 1.15 | 0.61 | 0.50 |  |  |  |  |  |  |  |  |  |  |  |  |  |  |  |  |  |  |  |  |
| 14 | *Echis coloratus* | ECO | 0.77 | 0.29 | 0.38 | 0.46 | 0.35 | 0.46 | 0.47 | 0.39 | 0.33 | 0.55 | 0.35 | 0.34 | 0.59 |  |  |  |  |  |  |  |  |  |  |  |  |  |  |  |  |  |  |  |
| 15 | *Enhydris enhydris* | EEN | 0.95 | 0.48 | 0.52 | 0.64 | 0.65 | 0.64 | 0.78 | 0.64 | 0.57 | 0.88 | 0.54 | 0.46 | 0.48 | 0.33 |  |  |  |  |  |  |  |  |  |  |  |  |  |  |  |  |  |  |
| 16 | *Epicrates maurus* | EMA | 0.86 | 0.37 | 0.61 | 0.77 | 0.58 | 0.77 | 0.46 | 0.79 | 0.63 | 0.73 | 0.63 | 0.37 | 0.65 | 0.40 | 0.80 |  |  |  |  |  |  |  |  |  |  |  |  |  |  |  |  |  |
| 17 | *Gonyosoma oxycephalum* | GOX | 1.34 | 0.46 | 0.89 | 1.00 | 0.63 | 1.00 | 0.90 | 0.90 | 0.87 | 1.14 | 0.80 | 0.49 | 0.89 | 0.48 | 0.63 | 0.71 |  |  |  |  |  |  |  |  |  |  |  |  |  |  |  |  |
| 18 | *Homalopsis buccata* | HBU | 1.03 | 0.53 | 0.70 | 0.81 | 0.78 | 0.81 | 0.97 | 0.86 | 0.71 | 1.06 | 0.66 | 0.47 | 0.66 | 0.40 | 0.85 | 0.94 | 0.83 |  |  |  |  |  |  |  |  |  |  |  |  |  |  |  |
| 19 | *Naja kaouthia* | NKA | 1.01 | 0.46 | 0.65 | 0.74 | 0.49 | 0.74 | 0.77 | 0.72 | 0.64 | 0.92 | 0.58 | 0.37 | 0.59 | 0.34 | 0.61 | 0.67 | 0.78 | 0.78 |  |  |  |  |  |  |  |  |  |  |  |  |  |  |
| 20 | *Naja siamensis* | NSI | 1.02 | 0.47 | 0.67 | 0.74 | 0.60 | 0.74 | 0.78 | 0.75 | 0.66 | 0.83 | 0.62 | 0.41 | 0.59 | 0.41 | 0.66 | 0.76 | 0.77 | 0.82 | 0.67 |  |  |  |  |  |  |  |  |  |  |  |  |  |
| 21 | *Oligodon fasciolatus* | OFA | 1.09 | 0.39 | 0.61 | 0.69 | 0.43 | 0.69 | 0.66 | 0.61 | 0.63 | 0.72 | 0.52 | 0.37 | 0.61 | 0.41 | 0.50 | 0.56 | 0.69 | 0.60 | 0.53 | 0.59 |  |  |  |  |  |  |  |  |  |  |  |  |
| 22 | *Ophiophagus hannah* | OHA | 1.12 | 0.44 | 0.77 | 0.89 | 0.54 | 0.89 | 0.84 | 0.83 | 0.77 | 0.94 | 0.67 | 0.39 | 0.73 | 0.38 | 0.64 | 0.73 | 0.92 | 0.81 | 0.68 | 0.75 | 0.59 |  |  |  |  |  |  |  |  |  |  |  |
| 23 | *Python bivittatus* | PBI | 1.36 | 0.48 | 1.01 | 1.44 | 0.65 | 1.44 | 1.45 | 1.37 | 1.10 | 1.23 | 1.03 | 0.46 | 1.11 | 0.57 | 1.36 | 1.08 | 1.25 | 1.73 | 1.18 | 1.23 | 0.87 | 1.36 |  |  |  |  |  |  |  |  |  |  |
| 24 | *Protobothrops flavoviridis* | PFL | 0.85 | 0.33 | 0.60 | 0.58 | 0.36 | 0.86 | 0.57 | 0.61 | 0.55 | 0.74 | 0.50 | 0.35 | 0.47 | 0.25 | 0.42 | 0.52 | 0.73 | 0.51 | 0.47 | 0.51 | 0.50 | 0.58 | 0.87 |  |  |  |  |  |  |  |  |  |
| 25 | *Pantherophis guttatus* | PGU | 1.06 | 0.44 | 0.68 | 0.81 | 0.60 | 0.81 | 0.77 | 0.76 | 0.70 | 1.01 | 0.60 | 0.41 | 0.71 | 0.39 | 0.60 | 0.73 | 0.80 | 0.78 | 0.64 | 0.69 | 0.55 | 0.75 | 1.28 | 0.52 |  |  |  |  |  |  |  |  |
| 26 | *Ptyas mucosa* | PMU | 1.52 | 0.48 | 0.97 | 1.24 | 0.73 | 1.24 | 1.02 | 1.01 | 1.03 | 1.05 | 0.88 | 0.46 | 0.94 | 0.49 | 0.79 | 0.86 | 1.13 | 0.98 | 0.92 | 0.92 | 0.76 | 1.07 | 1.73 | 0.76 | 1.01 |  |  |  |  |  |  |  |
| 27 | *Python regius* | PRE | 1.19 | 0.43 | 0.98 | 1.26 | 0.47 | 1.26 | 1.35 | 1.27 | 0.97 | 1.02 | 0.95 | 0.39 | 1.00 | 0.51 | 1.32 | 0.92 | 1.16 | 1.59 | 1.05 | 1.13 | 0.79 | 1.18 | 1.16 | 0.77 | 1.16 | 1.56 |  |  |  |  |  |  |
| 28 | *Thamnophis sirtalis* | TSI | 0.89 | 0.39 | 0.57 | 0.68 | 0.45 | 0.68 | 0.73 | 0.65 | 0.58 | 0.81 | 0.54 | 0.35 | 0.58 | 0.30 | 0.57 | 0.65 | 0.73 | 0.72 | 0.58 | 0.65 | 0.49 | 0.63 | 1.10 | 0.46 | 0.59 | 0.81 | 0.95 |  |  |  |  |  |
| 29 | *Vipera ammodytes* | VAM | 0.88 | 0.38 | 0.66 | 0.61 | 0.46 | 0.61 | 0.55 | 0.58 | 0.43 | 0.91 | 0.46 | 0.36 | 1.53 | 0.44 | 0.40 | 0.52 | 0.77 | 0.52 | 0.42 | 0.47 | 0.46 | 0.51 | 0.91 | 0.27 | 0.57 | 0.78 | 0.79 | 0.42 |  |  |  |  |
| 30 | *Vipera berus berus* | VBR | 0.97 | 0.44 | 0.74 | 0.78 | 0.53 | 0.78 | 0.63 | 0.66 | 0.53 | 0.94 | 0.55 | 0.40 | 0.76 | 0.38 | 0.45 | 0.56 | 0.84 | 0.57 | 0.52 | 0.56 | 0.56 | 0.66 | 0.97 | 0.37 | 0.63 | 0.89 | 0.87 | 0.49 | 0.55 |  |  |  |
| 31 | *Xenochrophis flavipunctatus* | XFL | 0.87 | 0.41 | 0.64 | 0.76 | 0.57 | 0.84 | 0.75 | 0.71 | 0.64 | 0.92 | 0.61 | 0.43 | 0.63 | 0.38 | 0.60 | 0.66 | 0.79 | 0.70 | 0.61 | 0.66 | 0.58 | 0.71 | 1.00 | 0.54 | 0.67 | 0.92 | 0.94 | 0.58 | 0.53 | 0.57 |  |  |
| 32 | *Xenopeltis unicolor* | XUN | 1.12 | 0.51 | 0.85 | 1.06 | 0.46 | 1.06 | 1.05 | 1.03 | 0.84 | 1.11 | 0.82 | 0.47 | 0.96 | 0.49 | 0.85 | 0.73 | 0.85 | 1.01 | 0.92 | 0.92 | 0.75 | 0.96 | 1.08 | 0.69 | 1.00 | 1.17 | 0.98 | 0.80 | 0.76 | 0.83 | 0.80 |  |
