# Supplementary figures and images for: Existence of Bov-B LINE Retrotransposons in Snake Lineages Reveals Recent Multiple Horizontal Gene Transfers with Copy Number Variation
Source: Genes (Basel). 2020 Oct 22;11(11):1241. doi: 10.3390/genes11111241 (PMC7716205; doi:10.3390/genes11111241)

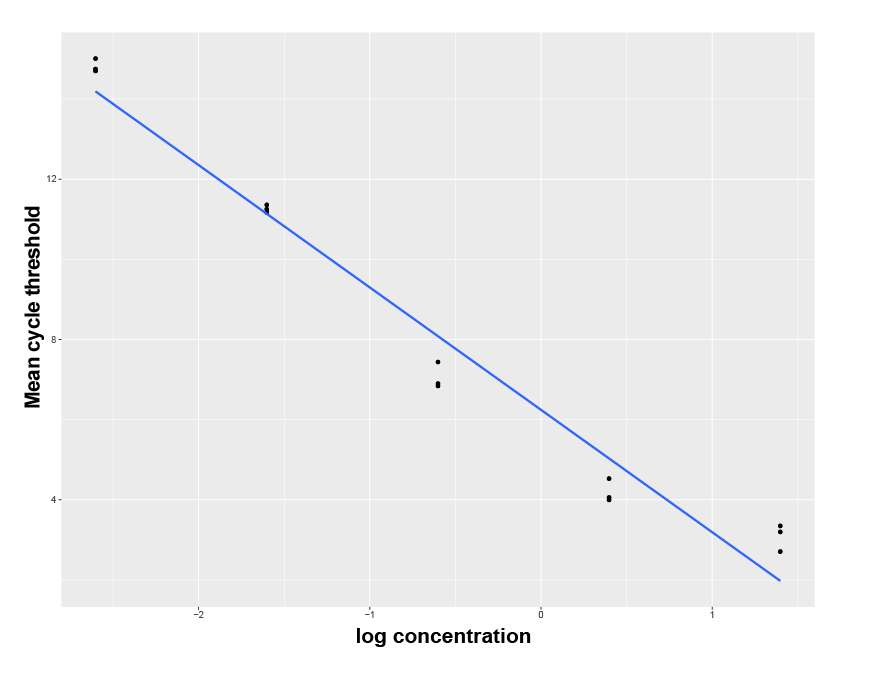

Supplement: Supplementary file 1 [file genes-11-01241-s001.zip › supplementary figure/Figure S1.jpg]

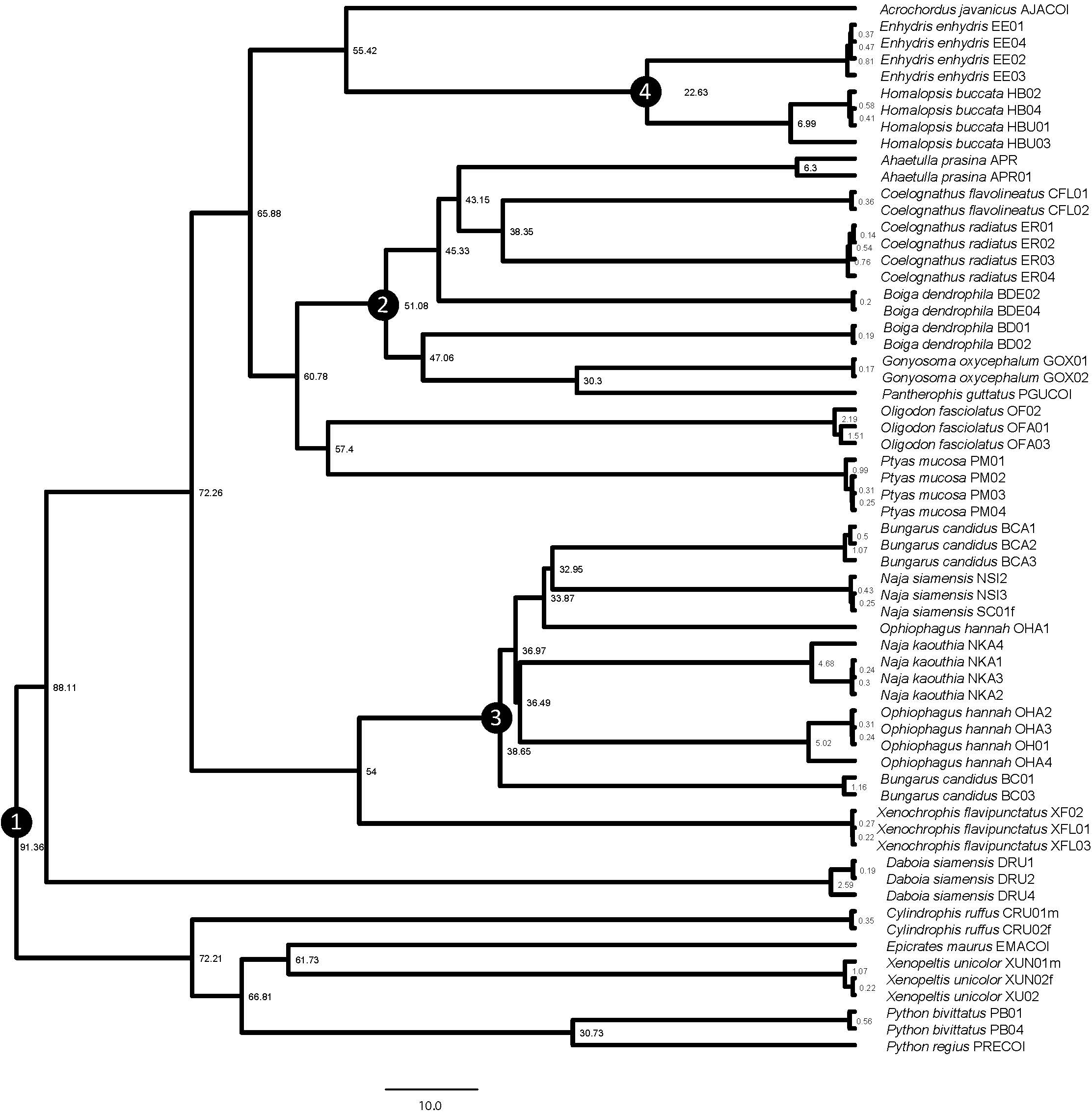

Supplement: Supplementary file 1 [file genes-11-01241-s001.zip › supplementary figure/Figure S2.jpg]

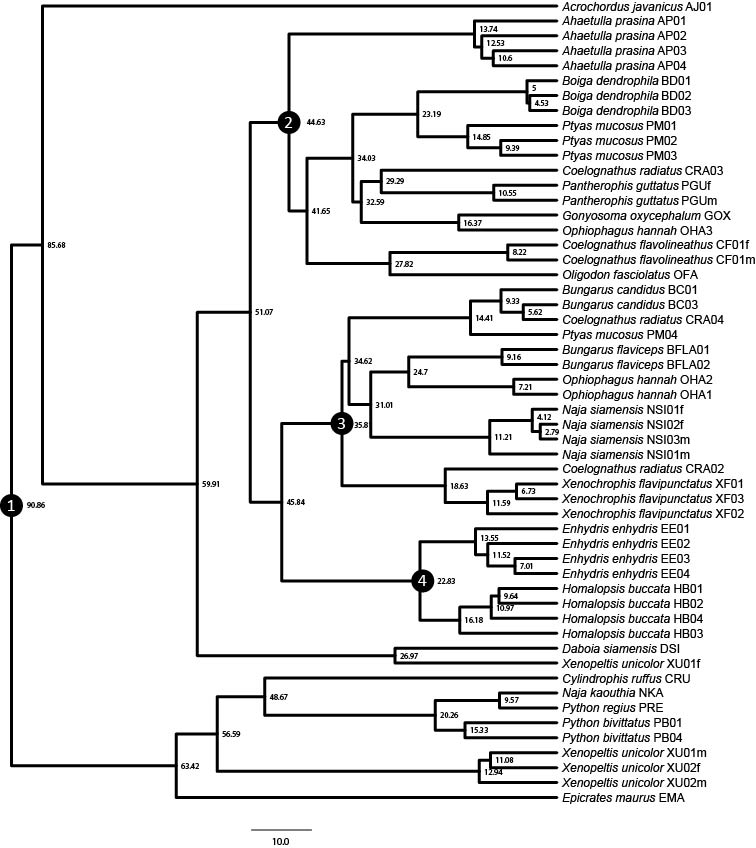

Supplement: Supplementary file 1 [file genes-11-01241-s001.zip › supplementary figure/Figure S3.jpg]
